# The 82-plex plasma protein signature that predicts increasing inflammation

**DOI:** 10.1038/srep14882

**Published:** 2015-10-08

**Authors:** Martin Tepel, Hans C. Beck, Qihua Tan, Christoffer Borst, Lars M. Rasmussen

**Affiliations:** 1 Department of Nephrology, Odense University Hospital, and University of Southern Denmark, Institute of Molecular Medicine, Cardiovascular and Renal Research, Institute of Clinical Research; 2 Department of Clinical Biochemistry and Pharmacology, Centre for Individualized Medicine in Arterial Diseases (Odense University Hospital), and Centre for Clinical Proteomics (Odense University Hospital/University of Southern Denmark); 3 Department of Epidemiology, Biostatistics and Biodemography, Institute of Public Health; Unit of Human Genetics, Institute of Clinical Research, University of Southern Denmark.

## Abstract

The objective of the study was to define the specific plasma protein signature that predicts the increase of the inflammation marker C-reactive protein from index day to next-day using proteome analysis and novel bioinformatics tools. We performed a prospective study of 91 incident kidney transplant recipients and quantified 359 plasma proteins simultaneously using nano-Liquid-Chromatography-Tandem Mass-Spectrometry in individual samples and plasma C-reactive protein on the index day and the next day. Next-day C-reactive protein increased in 59 patients whereas it decreased in 32 patients. The prediction model selected and validated 82 plasma proteins which determined increased next-day C-reactive protein (area under receiver-operator-characteristics curve, 0.772; 95% confidence interval, 0.669 to 0.876; P < 0.0001). Multivariable logistic regression showed that 82-plex protein signature (P < 0.001) was associated with observed increased next-day C-reactive protein. The 82-plex protein signature outperformed routine clinical procedures. The category-free net reclassification index improved with 82-plex plasma protein signature (total net reclassification index, 88.3%). Using the 82-plex plasma protein signature increased net reclassification index with a clinical meaningful 10% increase of risk mainly by the improvement of reclassification of subjects in the event group. An 82-plex plasma protein signature predicts an increase of the inflammatory marker C-reactive protein.

C-reactive protein is an acute-phase-reactant synthesized by hepatocytes and an exquisitely sensitive systemic marker of tissue damage, tissue ischemia, infection, and inflammation^1^. C-reactive protein levels rise rapidly in response to inflammatory stimuli, including several cytokines and tumor necrosis factor alpha. Determination of C-reactive protein is established after renal transplantation^2^,^3^,^4^,^5^,^6^.

The impact of identical levels of C-reactive protein may be interpreted differently, whether the concentrations show an upward or downward trend. Until now, the day-to-day development of C-reactive protein cannot be determined at the time of its measurement in plasma. However, human plasma contains more than 10,000 different proteins whereof approximately 1,200 have been quantified (http://www.plasmaproteomedatabase.org/) including other acute-phase reactants, proteinase inhibitors, coagulation proteins, complement proteins, and transport proteins^1^,^7^. Importantly, many proteins display unique sensitivity, response speed, and dynamic range to the inflammatory stimulus. Therefore it is probable to determine the specific plasma protein signature which predicts an increased inflammatory response.

Modern proteome analysis and bioinformatics may provide a way to identify the underlying plasma protein signature, but that has not been proven yet. We hypothesized that it is possible to define a plasma protein signature that predicts the increase of next-day C-reactive protein, i.e. an increase of C-reactive protein from index day to next-day. Plasma proteome analysis has been attempted to predict cancer incidence and mortality. A few publications using peptide pattern recognition to diagnose prostate or ovarian cancer spurred enthusiasm, but no protein identification and no validation was done and findings were difficult to reproduce^8^,^9^,^10^. The technical and bioinformatics armament of proteome analysis have however developed quickly over the last years.

In this study, we present for the first time an 82-plex plasma protein signature that predicts the increase of the inflammatory marker C-reactive protein. Our approach in plasma proteomics is to do quantitative proteome analysis in individual samples from a substantial number of individuals to test if relevant predictors for clinical variables can be developed in a real-world routine after kidney transplantation. We used nano-Liquid-Chromatography-Tandem-Mass-Spectrometry (nano-LC-MSMS), performed the quantitative proteome analysis on individual samples in a cohort of kidney transplanted patients and validated results by modern bioinformatics and statistical analysis.

## Materials and Methods

### Study Population

The study protocol was in accordance with the ethical standards of the Declarations of Helsinki and Istanbul (The clinical and research activities being reported are consistent with the Principles of the Declaration of Istanbul as outlined in the ‘Declaration of Istanbul on Organ Trafficking and Transplant Tourism’) and was approved by the local ethics committee (Den Videnskabsetiske Komite for Region Syddanmark, Projekt-ID: 8-20100098). Patients who were at least 18 years old and who were scheduled to receive donor kidney transplants were recruited. Written informed consent was obtained from all patients before entry into the study. Baseline characteristics and information on organ procurement were obtained from medical records and comprised personal history and previous history of renal disease. Induction therapy, immunosuppressive therapy, and concomitant medications were all made by the clinicians according to local protocols. Immunosuppressive regime consisted of basiliximab, tacrolimus, and mycophenolate mofetil. Recipients with ABO-incompatible donor received rituximab and immunabsorption before transplantation as well as tacrolimus, mycophenolate mofetil, and prednisolone. We also investigated when incident kidney transplant recipients were discharged from the hospital. Clinicians were unaware of results from proteomic analyses.

### Sample preparation

We investigated whether a specific plasma protein signature can predict the increase of the inflammation marker C-reactive protein from index day to next-day in patients after kidney transplantation. The samples of the index day were taken during hospitalization at a median of 1 day (IQR, 1 to 2) after incident renal transplantation. Patients were asymptomatic after transplantation. Neither fever nor septicemia was observed at the time plasma samples were observed in the patients after transplantation. Plasma proteome was performed at dayX using nano-LC-MSMS. In addition, C-reactive protein levels were measured at dayX and at dayX + 1 using an immune-assay. The term “dayX + 1” is called “next-day C-reactive protein”

Plasma samples were diluted 10-fold with phosphate-buffered saline followed by the determination of the protein concentration using the Pierce BCA protein assay kit (Thermo Scientific, Sunnyvale, CA, USA). Plasma proteins were precipitated by adding a volume equivalent to 100 μg protein (approximately 15 μL) and diluting with 500 μL ice cold acetone followed by incubation at −20 °C for 1 hour and centrifugation (20.000g, 4 °C, 10 minutes). The supernatant was discarded and the remaining protein pellet was re-dissolved by the addition of 100 μL digestion buffer (0.5 M TEAB). Proteins were then reduced by adding dithiotretiol to a final concentration of 5 mmol/L followed by incubation at 50 °C for 30 minutes. The reduced sulfhydryl groups were then blocked by the addition of iodoacetamide to a final concentration of 15 mmol/L and incubation at room temperature in darkness for 30 min. Tryptic digestion was performed by the addition of trypsin (Promega, Madison, WI, USA). The protein-to-trypsin-ratio was 50 to 1 (w/w).

### Stable isotop labeling of plasma samples with isobaric tags for relative and absolute quantitation (iTRAQ)

A 5 μg fraction of the tryptic digest were collected from the samples for labeling with the four-plex iTRAQ kit (AB SCIEX, Framingham, MA, USA). The content of each iTRAQ reagent vial was diluted in ethanol delivered with the iTRAQ kit and one twentieth of each reagent vial was used to label a 5 μg fraction of a plasma sample. The labeling of the plasma samples was done as follows: iTRAQ reagent 114, laboratory control plasma sample, containing pooled plasma from 100 healthy individuals; iTRAQ reagent 115, pool of all 91 patient samples; iTRAQ reagent 116 and 117, individual patient samples. The labeled samples were pooled in equal ratios, i.e. every sample set contained two patient samples, a pool of all patient samples and a healthy control sample. The samples were dried in a vacuum centrifuge and re-dissolved in 50 μL of a 0.1% trifluoroacetic acid solution, purified using microcolumn packed with reversed phased material, containing equal w/w amounts of Poros R2 and Oligo R3 material. Bound peptides were eluted with 16 μL hydrophilic interaction chromatography buffer A, containing 90% acetonitrile and 0.1% TFA.

### Fractionation of samples by hydrophilic interaction chromatography (HILIC)

The resulting peptide mixtures (91 patient samples) were further fractionated using HILIC (hydrophilic interaction liquid interaction chromatography) using the fraction collection option of the Thermo/Dionex Ultimate 3000 nano/capillary HPLC system (Thermo Scientific, Sunnyvale, CA, USA). Briefly, the peptide samples were loaded onto a custom made HILIC column packed with TSKgel Amide-80 column material (3 μm bead size, 10 cm length, 300 μm ID, Tosoh Bioscience LLC, PA, USA) with a flow of 12 μL per minute of 90% buffer B (100% ACN and 0.1% TFA) and 10% buffer A (0.1% TFA) for 8.6 minutes followed by a decrease in the flow from 12 μL per minute to 6 μL per minute over 0.4 minutes. The gradient applied for peptide fractionation was as follows: 0 to 8.6 minutes (90% B); 8.6 to 9.0 minutes (90 to 85.5% B); 9 to 35 minutes (85.5 to 54% B); 35 to 39 minutes (54–10% B); 39 to 42 minutes (10% B); 42 to 43 minutes (10 to 90% B); 43 to 48 minutes (90% B). Fractions were collected every other minute from the 6th to the 22nd minute followed by the collection of a fraction from the 22nd to the 28th minute.

#### Nano-liquid-chromatography-tandem-mass-spectrometry (nano-LC-MSMS)

The collected fractions were analyzed by nano-Liquid-Chromatography-Tandem-Mass-Spectrometry (nano-LC-MSMS) analysis using a Dionex Ultimate 3000 nano HPLC coupled to a Thermo Scientific Orbitrap Q-Exactive mass spectrometer (Thermo Scientific, Bremen, Germany). Briefly, the samples (5 μL) were loaded onto a custom made fused capillary pre-column (2 cm length, 360 μm OD, 75 μm ID) with a flow of 5 μL per minutes for 7 minutes. Trapped peptides were separated on a custom made fused capillary column (20 cm length, 360 μm outer diameter, 75 μm inner diameter) packed with ReproSil Pur C18 3-μm resin (Dr. Maish, Ammerbuch-Entringen, Germany) with a flow of 250 nL per minute using a linear gradient from 95% solution A (0.1% formicacid) to 30% B (100% Acetonitrile in 0.1% formicacid) over 33 minutes or 54 minutes followed by 6 minutes at 90% B and 5 minutes at 98% A. Mass spectra were acquired in positive ion mode applying automatic data-dependent switch between one Orbitrap survey MS scan in the mass range of 400 to 1500 m/z followed by HCD fragmentation and Orbitrap detection of the ten or twelve most intense ions observed in the MS scan. Target value in the Orbitrap for MS scan was 1,000,000 ions at a resolution of 70,000 at m/z 200. Fragmentation in the HCD cell was performed at normalized collision energy of 30 eV. Ion selection threshold was set to 25,000 to 160,000 counts. Selected sequenced ions were dynamically excluded for 45 seconds.

### Processing of proteome data and protein quantification

A combined MASCOT-SEQUEST search was performed where peak lists (mgf files) were processed using the Proteome Discoverer 1.4, version 1.4.0.288. The search parameters were set to: MS accuracy 10 ppm, MSMS accuracy 0.1 Da for HCD data, with two missed cleavages allowed, fixed modification of cystein blocked with carbamidomethyl, lysine and N-terminal iTRAQ, and variable modifications; methionine oxidation, and deamidated asparagines. Tandem mass spectra were searched against the Swissprot database restricted to humans downloaded October 2012 from http://www.ebi.ac.uk. Proteins were inferred on a basis of at least two unique peptides identified with a high confidence. False discovery rates were obtained using Percolator selecting identification with a q-value equal or less than 0.01. iTRAQ quantification was performed using Proteome Discoverer with reporter ion area integration within a 20 ppm window. Ratios were normalized against the median peptide ratio. The data used for statistical analysis were ratios of diseased individuals (reporter ions 116 and 117) versus the plasma control (reporter ion 114: pool of healthy individuals). The ratios of 115 vs. 114 were used as a measure for the technical variability of the method that was calculated to be less than 10%.

### Measurement of C-reactive protein concentrations

The immune-assay based plasma C-reactive protein quantification was routinely conducted at the index day and the next-day on an Architect C8000/c16000 system (Abbot Diagnostics, IL, USA) using the C-reactive protein Vario kit (reference number: 6K26-30/6K26-41, SENTINEL, CH. SPA) in the concentration range from 0.2 to 480 mg/L. The coefficient of variation of the method was less than 6 percent.

### Data analysis and statistics

Continuous data are presented as median and interquartile range (IQR). We stratified the cohort into groups with increased and non-increased next-day C-reactive protein levels. The data distribution was tested using Kolmogorof-Smirnov test. Non-parametric Mann-Whitney test was used to detect differences between the groups. Frequency counts were calculated for categorical data. Differences in these categorical variables between the groups were analyzed by Fisher’s exact text. Associations between variables were determined using non-parametric Spearman correlation. We performed receiver operating characteristic (ROC) curve analysis for this prediction model for increased next-day C-reactive protein.

Univariable and multivariable logistic regression analyses for observed increased next-day C-reactive protein in incident kidney transplant recipients were performed, testing for 82-plex protein signature, donor age, donor gender, donor status, recipient age, recipient gender, duration of dialysis before transplantation, use of methylprednisolon, and C-reactive protein level at the same day. Multivariable models were constructed with backward variable selection, using P < 0.05 for variable retention.

### Model development and cross validation

We followed the strategy of PAM (Prediction Analysis of Microarray at http://statweb.stanford.edu/~tibs/PAM/) for building our prediction model using support vector machines (SVMs). A SVM is a supervised machine learning model that, with its associated learning algorithm, maps samples in space such that samples of separate categories are divided as wide as possible. We used the “radial” kernel, the default kernel of the R package e1071 for SVM. Our classification process uses a double-cross-validation scheme. The internal loop is to train an optimal prediction model for use in the outer loop. We stick to the default kernel with consideration of generalization of the prediction model. In fact, we experimented with other kernels during model fitting and found that the default “radial” kernel gave the best performance. SVM was used for the prediction of next-day increase of C-reactive protein.

We calculated Pearson correlation coefficients for plasma protein concentrations of each protein with changes of next-day C-reactive protein in all 91 patients for use of feature or protein selection in the model building process. Similar to PAM, our feature selection was done by recursively shrinking correlation smaller than a predefined threshold to zero and using the remaining subset of proteins for prediction model building. The threshold that corresponds to the lowest prediction error was taken as the optimal cut-off for defining the final sub-set of proteins for training the classification model using the support vector machine. Performance of the classification model was assessed by leave-one-out cross validation (LOOCV). LOOCV used a single sample from the original 91 patients as the validation data, and the remaining 90 observations as the training data. This was repeated such that each of the 91 patients was used once as the validation data. Based on the prediction result for each patient by LOOCV, we calculated the proportion of patients whose next-day C-reactive protein levels were correctly predicted as increased (sensitivity) or as non-increased (specificity). The overall mean accuracy of the prediction model was calculated as the proportion of all correct predictions. C-reactive protein levels at the same day were not included in the prediction model.

### Integrated discrimination improvement (IDI) and net reclassification improvement (NRI)

The integrated discrimination improvement (IDI) and net reclassification improvement (NRI), have been rapidly adopted to quantify the added value of a biomarker to an existing test^11^,^12^,^13^. To assess the independent predictive ability of the 82-plex plasma protein signature relative to specified predictors of increasing C-reactive protein, i.e. donor age, multivariable logistic regression modeling was used without and with 82-plex plasma protein signature.

The category-free net reclassification index (cfNRI) and integrated discrimination index (IDI) were calculated to assess the incremental predictive ability of the 82-plex plasma protein signature. The cfNRI considers whether each individual moves up (to higher) or down (to lower) in individual calculated risk^11^,^12^,^13^. The integrated discrimination index (IDI) was used to quantify the actual change in calculated risk for each individual for those with and those without events^11^,^12^,^13^. For calculating category-free net reclassification index (cfNRI) we used the following equations:

For patients with events improved reclassification was the difference between the percentage of patients who were reclassified as being at higher risk and the percentage of patients who were reclassified to lower risk.

cfNRIevents = (number events with increased predicted risk after addition of the 82-plex plasma protein signature to the model/number events) minus (number events with decreased predicted risk after addition of the 82-plex plasma protein signature to the model/number events).

For patients without events, improved reclassification was the difference between the percentage of patients who were reclassified to lower risk and the percentage of patients who were reclassified to higher risk.

cfNRIno-events = (number no-events with decreased predicted risk after addition of the 82-plex plasma protein signature to the model/number no-events) minus (number no-events with increased predicted risk after addition of the 82-plex plasma protein signature to the model/number no-events).

The total cfNRI was the sum of correct reclassification among patients with and without event:

Total cfNRI = cfNRIevents + cfNRIno-events

The maximum total cfNRI is 200% (predicted risks for all subjects with events are increased, and all subjects without events are decreased).

IDIevents = (Sum of propability of event after addition of the 82-plex plasma protein signature to the model/number events) minus (Sum of propability of event of predicted risk without addition of the 82-plex plasma protein signature to the model/number events).

IDIno-events = (Sum of propability of event without addition of the 82-plex plasma protein signature to the model/number no-events) minus (Sum of propability of event of predicted risk after addition of the 82-plex plasma protein signature to the model/number no-events).

The total IDI was the sum of correct reclassification among patients with and without event:

Total IDI = IDIevents + IDIno-events

Data analysis was powered by the statistical package “R” (http://www.r-project.org/), GraphPad prism software (version 5.0, GraphPad Software, San Diego, CA, USA), and SPSS for windows (version 15.0; SPSS, Chicago, IL, USA). All statistical tests were two-sided. Two-sided P-values less than 0.05 were considered to indicate statistical significance.

## Results

### Patients characteristics and outcome

The proteome analysis was performed in 91 incident kidney transplant recipients. 53 transplant recipients were male (58%), and 38 were female (42%). Median age of recipients was 51 years (Interquartile range (IQR), 45 to 59 years). The cause of chronic kidney disease was diabetic nephropathy in 13 cases (14%), hypertensive nephropathy in 9 cases (10%), chronic glomerulonephritis in 32 cases (35%), polycystic kidney disease in 17 cases (19%), and other/unknown in 20 cases (22%). The number of patients with second or more transplants was 15 (16%). Median time on dialysis before transplantation was 12 months (IQR, 1 to 50 months). 20 patients (22%) were smokers, 69 patients (76%) had hypertension, and 12 patients (13%) had a history of cardiovascular events. 51 patients (56%) received kidneys from living donors, and 40 patients (44%) from deceased donors. All patients (100%) received calcineurin inhibitors and mycophenolate mofetil. 29 patients (32%) received methylprednisolone. The routinely measured median C-reactive protein was 35 mg/L (IQR, 16 to 67 mg/L). Normal reference values for C-reactive protein were less than 6 mg/L. In 59 patients next-day C-reactive protein increased by 46 mg/L (IQR, 25 to 82 mg/L) whereas in 32 patients it decreased by 5 mg/L (IQR, 21 to 3 mg/L). The clinical characteristics of patients and their allografts are shown in Table 1. In univariable analyses, kidney transplant recipients who showed increased next-day C-reactive protein had higher donor age and shorter time on dialysis. Absolute C-reactive protein concentrations were not significantly different in patients with increased or non-increased next-day C-reactive protein (P = 0.329).

### Plasma proteome predicts increased next-day C-reactive protein

**Figure 1:**
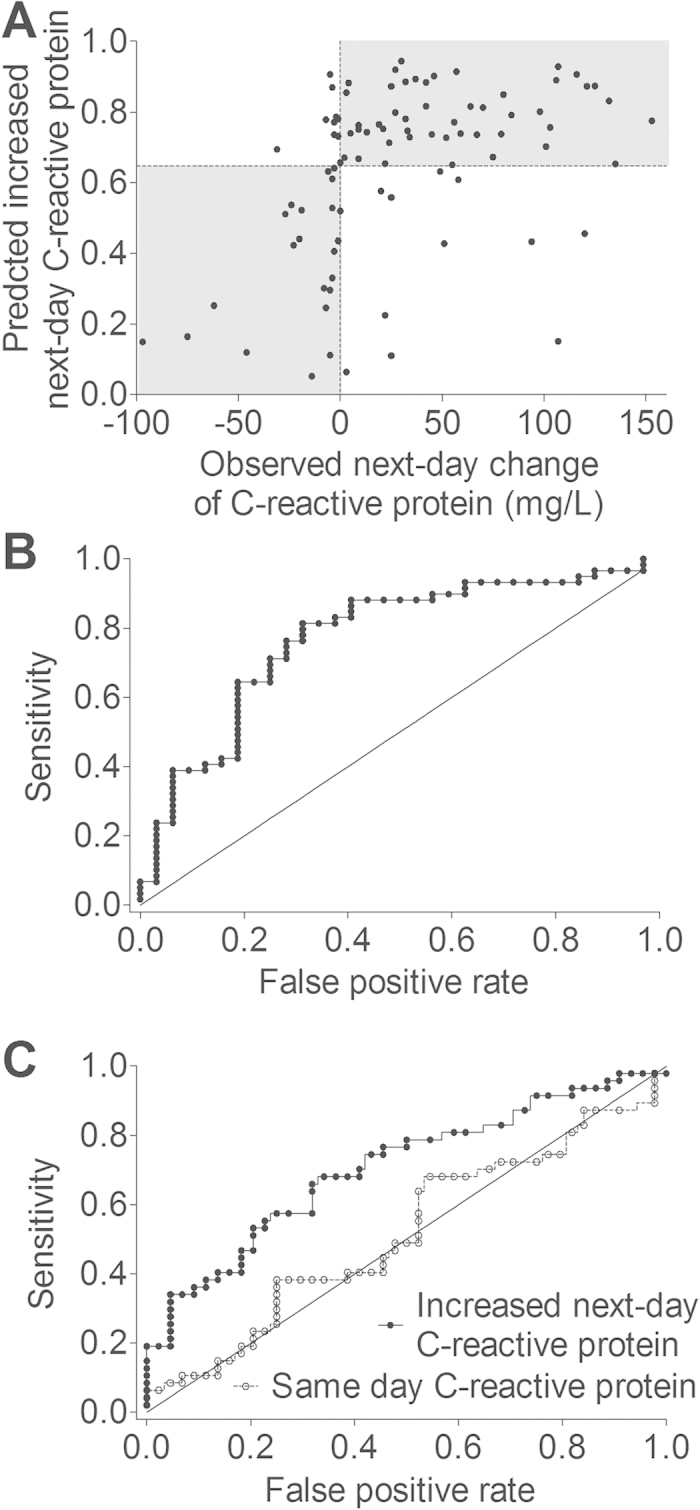
(**A**) 82-plex protein signature determines increased next-day C-reactive protein. The probability of increased next-day C-reactive protein according to the prediction model is shown against observed next-day C-reactive protein changes (in mg/L) in 91 incident kidney transplant recipients. The horizontal line cuts the probability at 0.648 which is the proportion of 59 patients with observed increased next-day C-reactive protein out of 91 patients. Patients above the line are predicted as with increased (81% correct, sensitivity) and patients below the line as with non-increased (69% correct, specificity) next-day C-reactive protein. (**B**) Receiver-operator-characteristics curve for prediction of increased next-day C-reactive protein from 82-plex protein signature. The area under curve was 0.772 (95% confidence interval, 0.669 to 0.876; P < 0.0001). (**C**) Receiver-operator-characteristics curve for hospitalization in 91 incident kidney transplant recipients. Filled circles indicate increased (vs. non-increased) next-day C-reactive protein determined by 82-plex protein signature (area under curve, 0.706; 95% confidence interval, 0.599 to 0.812; P = 0.0007). Open circles indicate C-reactive protein level above (vs. below) median value determined at the same day proteomic analysis were performed by immunoassay, i.e., 35mg/L (area under curve, 0.517; 95% confidence interval, 0.397 to 0.637; P = 0.775).

Using nano-Liquid-Chromatography-Tandem-Mass-Spectrometry (nano-LC-MSMS) we measured 359 plasma proteins simultaneously (Supplemental Table 1). To define a protein signature that predicted increased next-day C-reactive protein a support vector machine based prediction model using leave-one-out cross validation and recursive shrinkage was built. Figure 1A shows the prediction of increased next-day C-reactive protein obtained from 82-plex protein signature. The prediction model selected 82 plasma proteins which determined increased next-day C-reactive protein with a sensitivity of 81% and a specificity of 69%. The overall accuracy was 77%, i.e. 70 out of 91 patients were correctly predicted. The receiver-operator-characteristics curve for this prediction model is shown in Fig. 1B. The area under curve was 0.772 (95% confidence interval, 0.669 to 0.876; P < 0.0001. This area obtained from the 82-plex protein signature was higher than that obtained from each of 82 plasma proteins (Table 2). For comparison, neither hemoglobin concentrations (area under curve, 0.599; 95% confidence interval, 0.477 to 0.720; P = 0.121), nor lymphocyte counts (area under curve, 0.514; 95% confidence interval, 0.385 to 0.642; P = 0.832) predicted increased next-day C-reactive protein. We did not observe any association of the protein signature with recipient age (Spearman r = −0.103), body mass index (Spearman r = 0.069), systolic blood pressure (Spearman r = 0.058), diastolic blood pressure (Spearman r = 0.102), hemoglobulin (Spearman r = 0.227), or leukocyte count (Spearman r = −0.042).

Next we evaluated whether 82-plex protein signature was able to predict outcome and outperform routine clinical procedures. Incident kidney transplant recipients were discharged from the hospital at median 7.0 days (IQR, 6.0 to 8.0 days). It should be noted that median hospitalization was longer in patients with increased vs. non-increased next-day C-reactive protein (7 days; IQR, 6 to 10 days; vs. 5.5 days; IQR, 4.25 to 7 days; P = 0.0003). Receiver-operator-characteristics curves showed that increased next-day C-reactive protein determined by the 82-plex protein signature predicted longer hospitalization (area under curve, 0.706; 95% confidence interval, 0.599 to 0.812; P = 0.0007), whereas C-reactive protein determined at the same day was not predictive (area under curve, 0.517; 95% confidence interval, 0.397 to 0.637; P = 0.775; Fig. 1C). These findings indicate that knowledge of the 82-plex protein signature was able to outperform routine clinical procedures and may improve patient care.

Univariable and multivariable logistic regression analyses for increased next-day C-reactive protein are given in Table 3. Multivariable logistic regression showed that only 82-plex protein signature (P < 0.001) and older donor age (P = 0.003) were associated with observed increased next-day C-reactive protein.

**Figure 2:**
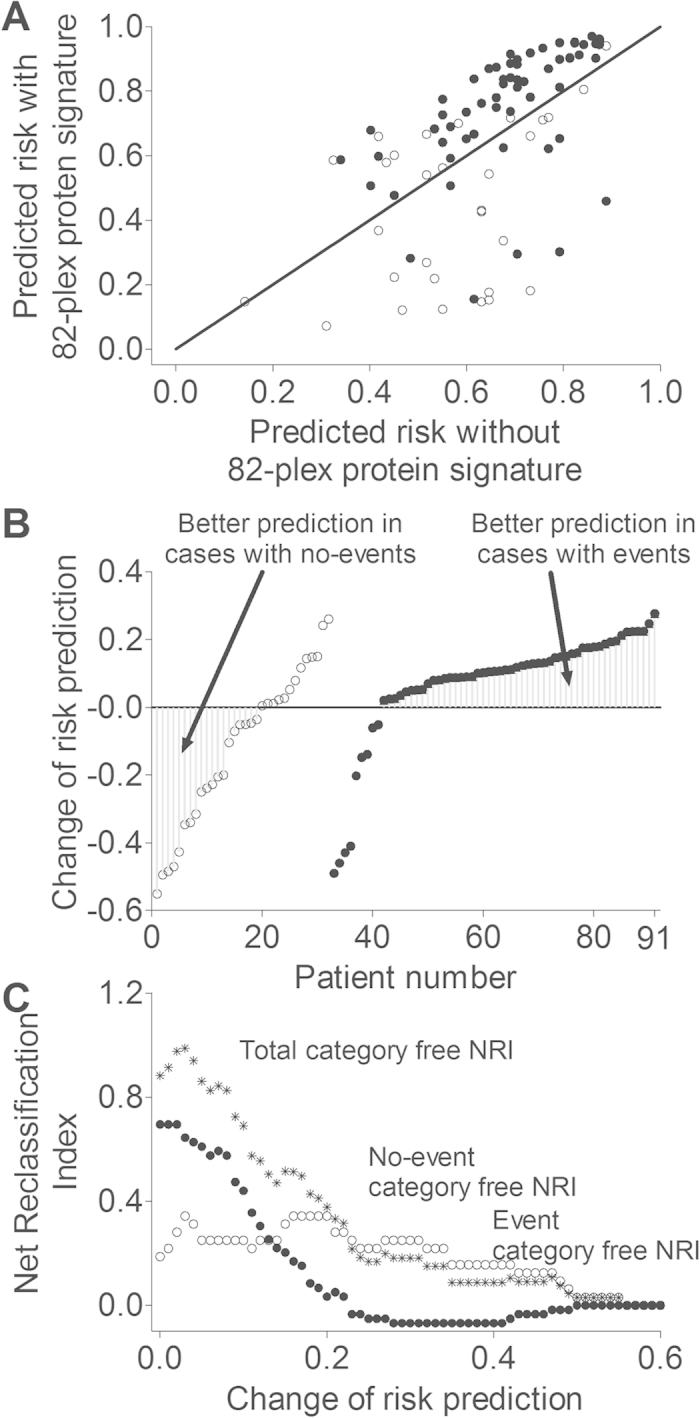
(**A**) Dot plot showing the Reclassification of patients with events (filled circles) and patients with no-events (open circles) after the addition of 82-plex protein signature to a model for prediction of risk for increased next-day C-reactive protein in incident patients after renal transplantation. The line of identity is given. (**B**) Dot plot showing the change of risk prediction for each patient with events (filled circles) and for each patient with no-events (open circles) after the addition of 82-plex protein signature to a model for prediction of risk for increased next-day C-reactive protein in incident patients after renal transplantation. Patients with better risk prediction as judged by observed outcome are indicated. (**C**) Category-free Net Reclassification Index (cfNRI) for total subjects (asterixes), subjects with events (filled circles) and subjects with no-events (open circles) as calculated according to the change of risk prediction (threshold value). The addition of the 82-plex protein signature increases the reclassification of the total group of subjects, i.e. reclassification of subjects in the event group to higher risk and reclassification of subjects in the no event group to lower risk. The graph indicates that an increased Net Reclassification Index at a clinical meaningful increase of risk by 10% is mainly driven by the improvement of reclassification of subjects in the event group.

To further describe the ability of 82-plex protein signature to risk-stratify patients beyond classical clinical risk prediction model, category-free net reclassification index (cfNRI) and integrated discrimination index (IDI) after addition of the 82-plex plasma protein signature to the model were calculated (Tables 4 and 5). The category-free net reclassification index provides a measure of the direction of change in reclassification that the 82-plex plasma protein signature adds to the clinical model, with results reported as proportions^11^,^12^,^13^. The integrated discrimination index provides information on both the direction and magnitude of mean change in predicted probabilities for events and nonevents when the 82-plex plasma protein signature was added to the clinical model^11^,^12^,^13^. The category-free net reclassification index improved with 82-plex plasma protein signature (total net reclassification index, 88.3%). That was mainly due to an improvement in the event group by 69.5% (Table 4). Furthermore, the integrated discrimination index showed an improvement by 0.3864 (95% CI, 0.3382 to 0.4345; Table 5). Figure 2 indicates that an increased net reclassification index at a clinical meaningful increase of risk by 10% is mainly driven by the improvement of reclassification of subjects in the event group.

As depicted in Supplemental Table 2 unadjusted Spearman correlation analyses of the 82 plasma proteins showed Spearman r values from minimum −0.405 (for angiotensinogen) to maximum 0.395 (for Ig_lambda_chain_V_III_region_LOI). Importantly, the correlation obtained from the 82-plex protein signature (Spearman r = 0.475, 95% CI, 0.292 to 0.624, P < 0.0001) was higher than those obtained from each 82 plasma proteins.

## Discussion

In our prospective study we present for the first time an 82-plex plasma protein signature that predicts the increase of the inflammatory marker C-reactive protein. The strength of the present study include the use of quantitative proteome analysis on individual samples as well as validation of the results using a support vector machine based prediction model using leave-one-out cross validation and recursive shrinkage. The novelty of our type of approach in plasma proteomics is to actually get it to work in a real-world routine after kidney transplantation. We also showed that the 82-plex plasma protein signature reclassified patients to a more appropriate level of risk using category-free net reclassification index and integrated discrimination index.

The present study indicated that quantitative plasma proteomics using nano-Liquid-Chromatography-Tandem-Mass-Spectrometry identifies a 82-plex protein signature which predicts increased next-day C-reactive protein in incident kidney transplant recipients. The validity of the present study is strengthened by using complementary approaches: First, receiver-operator-characteristics curve confirmed a significant power of our prediction model building based on plasma proteomics. Second, multivariable logistic regression showed that only 82-plex protein signature (P < 0.001) and older donor age (P = 0.003) were associated with increased next-day C-reactive protein. That multivariable logistic regression analysis also confirmed that our cohort includes typical incident kidney transplant recipients because it confirmed earlier findings that older donor age is associated with an increased inflammatory response. De Fijter *et al.* showed that kidneys from older donors show increased immunogenicity^14^. This may induce several proinflammatory cytokines, contributing to a general stereotyped response to tissue injury^15^,^16^. However, the demonstration of improved laboratory data impact and patient care after kidney transplantation by identification of the 82-plex protein signature using plasma proteomics is also novel for clinical routine.

Several biological mechanisms provide a plausible explanation for the causal role of the specific 82-plex protein signature and increased next-day C-reactive protein in incident kidney allograft recipients. Compared to C-reactive protein inflammatory stimuli may be detected earlier using proteomic measurements. Several cytokines causing increased C-reactive protein abundance showed increased levels after kidney transplantation. Cho *et al.* reported a 150 to 200 percent increase of several interleukins and tumor necrosis factor alpha during the postoperative days^17^. Because C-reactive protein levels start rising approximately 6 hours after an inflammatory stimulus, 1 we have chosen to identify a protein signature that predicts increased next-day C-reactive protein. Taking the rapid C-reactive protein kinetics into account, an increased next-day C-reactive protein obtained from proteome analysis defining the 82-plex protein signature represented the extent of the inflammatory stimulus. The 82-plex protein signature can be obtained at any time because it predicts increased next-day C-reactive protein but does not depend on absolute levels. Daily measurements of C-reactive protein have been introduced as a simple and sensitive method for detecting complications after renal transplantation in adult and pediatric transplant recipients^3^,^18^. Daily C-reactive protein measurements have been shown to help identification of renal allograft dysfunctions of different origins. However, C-reactive protein is not a specific marker to indicate rejection or infection or ischemia after kidney transplantation. C-reactive protein cannot discriminate the underlying causes^17^,^18^. In contrast, determination of increasing next-day C-reactive protein using the proteomic approach may superior to determination of same day C-reactive protein solely indicating upcoming inflammatory responses.

Is knowledge of the 82-plex protein signature superior to current methodology? 82-plex protein signature was also able to predict outcome and to outperform routine clinical procedures. The 82-plex protein signature predicted increased next-day C-reactive protein. We showed that increased next-day C-reactive protein determined by the 82-plex protein signature predicted longer hospitalization, whereas currently used measurement of C-reactive protein at the same day was not predictive. We observed the patients with increased next-day C-reactive protein showed longer hospitalization. This finding is probably due to the fact that an increasing C-reactive protein level indicates a complication after transplantation. Hence earlier detection using the proteomic approach would be beneficial. Studies in other diseases indicated that the knowledge of C-reactive protein development may also improve patient care. For example, a delayed normalization of C-reactive protein levels has been associated with a higher risk of having received inappropriate antibiotic treatment in patients with community-acquired pneumonia^19^. Repeated determinations of the C-reactive protein has been shown to be useful in the recognition of the individual clinical course, either improving or worsening, as well as the rate of improvement, in patients with severe community-acquired pneumonia^20^. Our study may indicate that quantitative proteome analysis of undepleted individual plasma samples may outperform simple measurements of single proteins. This development is parallel to a similar successful development is emerging concerning the use of multiplex tissue-RNA-profiling in the diagnosis and prognosis of cancer^21^.

It is known that glucocorticoids affect the gene expression, for example they may up-regulate phosphoenol pyruvate carboxykinase involved in regulating gluconeogenesis or secretory leukocyte protease inhibitor and the type II interleukin-1 receptor, which may contribute to anti-inflammatory properties^22^,^23^. However, in the present study, multivariable analyses showed that use of methylprednisolon did not affect observed increased next-day C-reactive protein in incident kidney transplant recipients.

A limitation of our study was that it was restricted to kidney transplant recipients. Further studies are needed to show whether the proteome signature may be predictive only in kidney patients or has wider applicability in predicting outcome hospitalization.

In summary, using quantitative plasma proteomics we determined an 82-plex plasma protein signature that is associated with an increase of the inflammatory marker C-reactive protein and thus give novel insights into the role of plasma proteome in the inflammatory response.

## Additional Information

**How to cite this article**: Tepel, M. *et al.* The 82-plex plasma protein signature that predicts increasing inflammation. *Sci. Rep.* **5**, 14882; doi: 10.1038/srep14882 (2015).

## Supplementary Material

Supplementary Information

## Figures and Tables

**Table 1:** Clinical characteristics of incident kidney transplant recipients and allografts.

**Characteristic**	All patients(n = 91)	Increased next-dayCRP (n = 59)	Non-increased next-dayCRP (n = 32)	P-value
Age of donor (years)	52 (45–60)	54 (49–62)	46 (40–53)	0.002
Number of male donor (percent)	41 (45%)	25 (42%)	16 (50%)	0.661
Number of deceased kidney donors (percent)	40 (44%)	19 (32%)	21 (66%)	0.004
Number of HLA mismatches (range, 0 to 6)	3 (2–4)	3 (2–4)	3 (3–4)	0.239
Age of recipient (years)	51 (45–59)	51 (45–58)	51 (45–61)	0.913
Number of male recipient (percent)	58 (60%)	38 (64%)	15 (47%)	0.123
Duration of dialysis before transplantation (months)	12 (1–50)	8 (0–35)	21 (8–79)	0.023
Body weight (kg)	79 (67–91)	80 (67–94)	73 (61–84)	0.056
Systolic blood pressure (mmHg)	150 (132–164)	150 (133–162)	149 (126–170)	0.787
Diastolic blood pressure (mmHg)	87 (76–94)	83 (76–94)	87 (77–94)	0.730
C-reactive protein level at the same day proteomic analysis were performed (mg/L)^a^	35 (16–67)	31 (16–59)	49 (13–97)	0.329

Data are shown for all patients and according to increased and non-increased next-day C-reactive protein (CRP) concentration. Continuous data are presented as median (interquartile range). Categorical data are presented as numbers (percent). Groups containing continuous date were compared using Mann-Whitney test, whereas groups containing categorical data were compared using Fisher’s exact text.

^a^Normal reference C-reactive protein levels are less than 6 mg/L.

**Table 2:** Receiver operating characteristic curve analyses of 82 plasma proteins for increased next-day C-reactive protein.

**Plasma protein**	**AUC**	**95% CI for AUC**	**P**
Ig_lambda_7_chain_C_region	0.519	0.379 to 0.660	0.766
Immunoglobulin_lambda_like_polypeptide_5	0.600	0.472 to 0.727	0.116
Ceruloplasmin	0.578	0.453 to 0.703	0.231
CD5_antigen_like	0.590	0.465 to 0.715	0.155
Ficolin_3	0.548	0.416 to 0.680	0.462
Ceruloplasmin	0.611	0.478 to 0.744	0.081
Prothrombin	0.721	0.612 to 0.831	0.0005
Haptoglobin	0.653	0.527 to 0.778	0.016
Haptoglobin_related_protein	0.590	0.454 to 0.725	0.171
Coagulation_factor_IX	0.537	0.410 to 0.663	0.579
Plasminogen	0.645	0.522 to 0.768	0.0.22
Antithrombin_III	0.688	0.499 to 0.804	0.003
Alpha_1_antitrypsin	0.663	0.538 to 0.788	0.010
Alpha_1_antichymotrypsin	0.681	0.571 to 0.791	0.004
Angiotensinogen	0.704	0.592 to 0.816	0.001
Alpha_2_macroglobulin	0.515	0.391 to 0.639	0.806
Complement_C3	0.581	0.455 to 0.707	0.203
Complement_C5	0.603	0.476 to 0.730	0.105
Low_molecular_weight_kininogen_1	0.595	0.468 to 0.723	0.144
Ig_kappa_chain_V_IV_region_Len	0.561	0.431 to 0.691	0.336
Ig_lambda_chain_V_I_region_NEW	0.691	0.577 to 0.804	0.003
Ig_lambda_chain_V_IV_region_Hil	0.631	0.510 to 0.752	0.041
Ig_heavy_chain_V_I_region_EU	0.622	0.493 to 0.751	0.057
Ig_heavy_chain_V_I_region_HG3	0.701	0.589 to 0.813	0.001
Ig_heavy_chain_V_III_region_CAM	0.628	0.505 to 0.721	0.051
Ig_kappa_chain_C_region	0.589	0.462 to 0.717	0.158
Ig_gamma_1_chain_C_region	0.655	0.532 to 0.778	0.014
Ig_gamma_3_chain_C_region	0.595	0.470 to 0.720	0.134
Ig_mu_chain_C_region	0.587	0.462 to 0.712	0.170
Ig_alpha_1_chain_C_region	0.564	0.445 to 0.684	0.308
Ig_alpha_2_chain_C_region	0.544	0.422 to 0.666	0.490
Ig_delta_chain_C_region	0.574	0.445 to 0.703	0.255
Apolipoprotein_A_I	0.585	0.459 to 0.711	0.180
Apolipoprotein_C_I	0.509	0.382 to 0.636	0.877
Apolipoprotein_C_II	0.509	0.391 to 0.627	0.881
Apolipoprotein_C_III	0.619	0.499 to 0.738	0.061
Fibrinogen_alpha_chain	0.603	0.485 to 0.721	0.104
Serum_amyloid_P_component	0.510	0.373 to 0.648	0.864
Complement_component_C9	0.632	0.498 to 0.767	0.037
Leucine_rich_alpha_2_glycoprotein	0.571	0.445 to 0.698	0.260
Alpha_1_acid_glycoprotein_1	0.561	0.428 to 0.694	0.335
Alpha_2_HS_glycoprotein	0.570	0.446 to 0.694	0.267
Transthyretin	0.568	0.444 to 0.692	0.280
Serum_albumin	0.678	0.562 to 0.794	0.005
Serotransferrin	0.551	0.432 to 0.669	0.420
Hemopexin	0.636	0.519 to 0.753	0.032
Plasma_kallikrein	0.527	0.400 to 0.655	0.665
Vitronectin	0.644	0.524 to 0.764	0.023
Apolipoprotein_B_100	0.596	0.470 to 0.722	0.129
Alpha_1B_glycoprotein	0.664	0.537 to 0.791	0.009
Apolipoprotein_D	0.515	0.395 to 0.636	0.806
Plasma_protease_C1_inhibitor	0.698	0.548 to 0.791	0.007
Complement_factor_I	0.623	0.494 to 0.753	0.053
Coagulation_factor_XIII_ B_chain	0.579	0.451 to 0.706	0.232
Tetranectin	0.509	0.375 to 0.642	0.892
Heparin_cofactor_2	0.669	0.542 to 0.796	0.007
Ig_heavy_chain_V_II_region_ARH77	0.597	0.471 to 0.722	0.127
Gelsolin	0.686	0.578 to 0.793	0.003
Apolipoprotein_A_IV	0.571	0.450 to 0.692	0.261
Complement_component_C8_alpha_chain	0.608	0.478 to 0.738	0.089
Complement_component_C8_beta_chain	0.548	0.419 to 0.677	0.449
Complement_component_C8_gamma_chain	0.519	0.391 to 0.647	0.764
Corticosteroid_binding_globulin	0.620	0.496 to 0.744	0.060
Alpha_2_antiplasmin	0.739	0.629 to 0.849	0.0001
Ig_lambda_2_chain_C_regions	0.671	0.550 to 0.792	0.007
Clusterin	0.544	0.421 to 0.667	0.485
Complement_component_C6	0.661	0.540 to 0.782	0.011
Alpha_1_acid_glycoprotein_2	0.582	0.454 to 0.709	0.197
Inter_alpha_trypsin_inhibitor_heavy_chain	0.651	0.527 to 0.775	0.017
Inter_alpha_trypsin_inhibitor_heavy_chain_H1	0.546	0.415 to 0.677	0.464
Pregnancy_zone_protein	0.580	0.450 to 0.710	0.221
C4b_binding_protein_beta_chain	0.535	0.395 to 0.675	0.584
Carboxypeptidase_N_subunit_2	0.546	0.412 to 0.680	0.467
Serum_paraoxonase/arylesterase_1	0.528	0.398 to 0.658	0.650
Serum_amyloid_A_4_protein	0.538	0.413 to 0.664	0.543
Insulin_like_growth_factor_binding_protein_complex_acid_labile_subunit	0.534	0.402 to 0.666	0.596
Lumican	0.524	0.398 to 0.650	0.700
Hemoglobin_subunit_beta	0.599	0.477 to 0.721	0.119
Hemoglobin_subunit_alpha	0.554	0.429 to 0.679	0.392
Ig_lambda_chain_V_III_region_LOI	0.704	0.585 to 0.823	0.001
Inter_alpha_trypsin_inhibitor_heavy_chain_H3	0.603	0.474 to 0.733	0.110
Hyaluronan_binding_protein_2	0.676	0.553 to 0.798	0.007

AUC indicates area under curve. CI indicates confidence interval.

**Table 3:** Univariable (upper panel) and multivariable (lower panel) logistic regression analyses for observed increased next-day C-reactive protein in incident kidney transplant recipients.

**Variable**	**Odds ratio**	**95% CI for Odds ratio**	**P**
82-plex protein signature	45.675	2.796 to 746.222	0.007
Donor age	1.098	1.032 to 1.169	0.003
Donor gender (0 = female; 1 = male)	0.641	0.210 to 1.962	0.436
Donor status (0 = LD; 1 = DD)	0.208	0.041 to 1.053	0.058
Recipient age	0.986	0.939 to 1.035	0.577
Recipient gender (0 = female; 1 = male)	0.812	0.252to 2.620	0.728
Duration of dialysis before transplantation (months)	0.994	0.980 to 1.008	0.414
Methylprednisolon (0 = no; 1 = yes)	0.271	0.062 to 1.177	0.081
C-reactive protein level at the same day proteomic analysis were performed	1.000	0.988 to 1.012	0.971
Variable	Odds ratio	95% CI for Odds ratio	P
82-plex protein signature	102.768	1.348 to 254.228	<0.001
Donor age	1.084	1.032 to 1.165	0.003

CI indicates confidence interval, LD indicates living donor, DD indicates deceased donor.

**Table 4:** The category-free net reclassification index (cfNRI) after addition of the 82-plex plasma protein signature to the model.

	Wholegroup n	Higher riskn (%)	Lower riskn (%)	cfNRI(%)	cfNRI (95% CI)
Events	59	50 (84.7%)	9 (15.3%)	69.5%	(50.7% to 92.6%)
Nonevents	32	13 (40.6%)	19 (59.4%)	18.8%	(12.4% to 27.3%)
Events plus Nonevents	91			88.3%	(44.8% to 173.7%)

Abbreviations: cfNRI, category-free Net Reclassification Index; CI, confidence interval.

**Table 5:** The integrated discrimination index (IDI) after addition of the 82-plex plasma protein signature to the model.

	Wholegroup n	**IDI (95% CI)**
Events	59	0.1282 (0.1108 to 0.1455)
Nonevents	32	0.2582 (0.1809 to 0.3354)
Events plus Nonevents	91	0.3864 (0.3382 to 0.4345)

Abbreviations: IDI, integrated discrimination index; CI, confidence interval.
