# Supplementary material for: The 82-plex plasma protein signature that predicts increasing inflammation
Source: Sci Rep. 2015 Oct 8;5:14882. doi: 10.1038/srep14882 (PMC4597208; doi:10.1038/srep14882)
Supplement: Supplementary Information [file srep14882-s1.doc]

**The 82-plex plasma protein signature that predicts increasing inflammation**

Martin Tepel^1^, Hans C. Beck^2^, Qihua Tan^3^, Christoffer Borst^1^, and Lars M. Rasmussen^2^

**Supplemental Material**

**Supplemental Table 1**: Characteristics of 359 quantified plasma proteins. Raw data are searched against the Swissprot database restricted to humans. Precision data for protein determination, i.e. protein coverage, number proteins, number unique peptides identified for each specific protein, number peptides identified for each protein, peptide spectrum matches (PSM), average Mascot score and average Sequest score are summarized values from all samples analyzed.

| **Accession** | **Description** | **Protein Cover-age** | **Number Proteins** | **Number Unique Peptides** | **Number Peptides** | **PSM** | **Average Mascot score** | **Average Sequest score** |
| --- | --- | --- | --- | --- | --- | --- | --- | --- |
| B9A064 | Immunoglobulin lambda-like polypeptide 5 OS=Homo sapiens GN=IGLL5 PE=2 SV=2 - [IGLL5_HUMAN] | 50.00 | 1 | 5 | 11 | 9141 | 2904.2 | 383.9 |
| O43866 | CD5 antigen-like OS=Homo sapiens GN=CD5L PE=1 SV=1 - [CD5L_HUMAN] | 51.87 | 1 | 18 | 18 | 969 | 310.6 | 34.8 |
| P00734 | Prothrombin OS=Homo sapiens GN=F2 PE=1 SV=2 - [THRB_HUMAN] | 63.02 | 2 | 38 | 38 | 6345 | 2327.9 | 323.4 |
| P00738 | Haptoglobin OS=Homo sapiens GN=HP PE=1 SV=1 - [HPT_HUMAN] | 75.86 | 3 | 21 | 39 | 33997 | 10339.1 | 1377.4 |
| P00747 | Plasminogen OS=Homo sapiens GN=PLG PE=1 SV=2 - [PLMN_HUMAN] | 75.56 | 1 | 55 | 55 | 8137 | 2615.4 | 339.5 |
| P01008 | Antithrombin-III OS=Homo sapiens GN=SERPINC1 PE=1 SV=1 - [ANT3_HUMAN] | 60.99 | 1 | 41 | 41 | 7948 | 2870.6 | 349.7 |
| P01009 | Alpha-1-antitrypsin OS=Homo sapiens GN=SERPINA1 PE=1 SV=3 - [A1AT_HUMAN] | 77.03 | 1 | 48 | 48 | 40984 | 13505.9 | 1846.3 |
| P01011 | Alpha-1-antichymotrypsin OS=Homo sapiens GN=SERPINA3 PE=1 SV=2 - [AACT_HUMAN] | 61.70 | 2 | 29 | 29 | 10282 | 4030.4 | 474.9 |
| P01019 | Angiotensinogen OS=Homo sapiens GN=AGT PE=1 SV=1 - [ANGT_HUMAN] | 25.77 | 1 | 10 | 10 | 2269 | 765.8 | 90.8 |
| P01023 | Alpha-2-macroglobulin OS=Homo sapiens GN=A2M PE=1 SV=3 - [A2MG_HUMAN] | 76.26 | 1 | 92 | 103 | 58113 | 19241.3 | 2452.3 |
| P01024 | Complement C3 OS=Homo sapiens GN=C3 PE=1 SV=2 - [CO3_HUMAN] | 92.06 | 1 | 165 | 165 | 58432 | 18715.0 | 2441.0 |
| P01031 | Complement C5 OS=Homo sapiens GN=C5 PE=1 SV=4 - [CO5_HUMAN] | 52.27 | 1 | 84 | 84 | 7464 | 2038.4 | 306.3 |
| P01598 | Ig kappa chain V-I region EU OS=Homo sapiens PE=1 SV=1 - [KV106_HUMAN] | 32.41 | 1 | 2 | 3 | 1006 | 447.9 | 50.3 |
| P01762 | Ig heavy chain V-III region TRO OS=Homo sapiens PE=1 SV=1 - [HV301_HUMAN] | 36.89 | 1 | 4 | 4 | 303 | 480.9 | 71.1 |
| P01766 | Ig heavy chain V-III region BRO OS=Homo sapiens PE=1 SV=1 - [HV305_HUMAN] | 34.17 | 1 | 2 | 3 | 1092 | 872.1 | 77.7 |
| P01834 | Ig kappa chain C region OS=Homo sapiens GN=IGKC PE=1 SV=1 - [IGKC_HUMAN] | 90.57 | 1 | 11 | 11 | 23152 | 9099.9 | 977.8 |
| P01857 | Ig gamma-1 chain C region OS=Homo sapiens GN=IGHG1 PE=1 SV=1 - [IGHG1_HUMAN] | 80.30 | 1 | 18 | 32 | 33361 | 11099.5 | 1419.8 |
| P01859 | Ig gamma-2 chain C region OS=Homo sapiens GN=IGHG2 PE=1 SV=2 - [IGHG2_HUMAN] | 75.77 | 1 | 12 | 25 | 25700 | 6990.8 | 1021.2 |
| P01860 | Ig gamma-3 chain C region OS=Homo sapiens GN=IGHG3 PE=1 SV=2 - [IGHG3_HUMAN] | 66.31 | 1 | 10 | 25 | 23530 | 6691.9 | 962.4 |
| P01861 | Ig gamma-4 chain C region OS=Homo sapiens GN=IGHG4 PE=1 SV=1 - [IGHG4_HUMAN] | 75.54 | 1 | 9 | 20 | 15250 | 4299.6 | 596.3 |
| P01871 | Ig mu chain C region OS=Homo sapiens GN=IGHM PE=1 SV=3 - [IGHM_HUMAN] | 61.50 | 2 | 17 | 30 | 11062 | 3454.5 | 429.0 |
| P01876 | Ig alpha-1 chain C region OS=Homo sapiens GN=IGHA1 PE=1 SV=2 - [IGHA1_HUMAN] | 66.01 | 1 | 8 | 19 | 10456 | 3623.2 | 466.3 |
| P02647 | Apolipoprotein A-I OS=Homo sapiens GN=APOA1 PE=1 SV=1 - [APOA1_HUMAN] | 82.77 | 1 | 42 | 42 | 23764 | 7100.5 | 877.2 |
| P02649 | Apolipoprotein E OS=Homo sapiens GN=APOE PE=1 SV=1 - [APOE_HUMAN] | 59.31 | 1 | 22 | 22 | 3520 | 1082.4 | 135.8 |
| P02652 | Apolipoprotein A-II OS=Homo sapiens GN=APOA2 PE=1 SV=1 - [APOA2_HUMAN] | 69.00 | 1 | 10 | 10 | 5978 | 1409.4 | 235.8 |
| P02654 | Apolipoprotein C-I OS=Homo sapiens GN=APOC1 PE=1 SV=1 - [APOC1_HUMAN] | 39.76 | 1 | 7 | 7 | 1931 | 459.4 | 73.7 |
| P02655 | Apolipoprotein C-II OS=Homo sapiens GN=APOC2 PE=1 SV=1 - [APOC2_HUMAN] | 56.44 | 1 | 5 | 5 | 1282 | 471.2 | 53.5 |
| P02656 | Apolipoprotein C-III OS=Homo sapiens GN=APOC3 PE=1 SV=1 - [APOC3_HUMAN] | 58.59 | 2 | 6 | 6 | 1865 | 903.6 | 97.7 |
| P02671 | Fibrinogen alpha chain OS=Homo sapiens GN=FGA PE=1 SV=2 - [FIBA_HUMAN] | 49.77 | 2 | 50 | 50 | 17707 | 5041.9 | 667.3 |
| P02675 | Fibrinogen beta chain OS=Homo sapiens GN=FGB PE=1 SV=2 - [FIBB_HUMAN] | 79.63 | 1 | 43 | 43 | 12700 | 3724.4 | 523.2 |
| P02679 | Fibrinogen gamma chain OS=Homo sapiens GN=FGG PE=1 SV=3 - [FIBG_HUMAN] | 74.83 | 4 | 37 | 37 | 13168 | 3989.8 | 529.9 |
| P02741 | C-reactive protein OS=Homo sapiens GN=CRP PE=1 SV=1 - [CRP_HUMAN] | 36.16 | 3 | 11 | 11 | 2052 | 565.4 | 79.6 |
| P02743 | Serum amyloid P-component OS=Homo sapiens GN=APCS PE=1 SV=2 - [SAMP_HUMAN] | 33.63 | 1 | 9 | 9 | 1304 | 454.2 | 48.9 |
| P02748 | Complement component C9 OS=Homo sapiens GN=C9 PE=1 SV=2 - [CO9_HUMAN] | 58.32 | 1 | 30 | 30 | 3869 | 1136.8 | 145.4 |
| P02749 | Beta-2-glycoprotein 1 OS=Homo sapiens GN=APOH PE=1 SV=3 - [APOH_HUMAN] | 71.30 | 1 | 24 | 24 | 6658 | 1653.3 | 301.7 |
| P02750 | Leucine-rich alpha-2-glycoprotein OS=Homo sapiens GN=LRG1 PE=1 SV=2 - [A2GL_HUMAN] | 59.94 | 1 | 18 | 18 | 2684 | 893.0 | 109.5 |
| P02753 | Retinol-binding protein 4 OS=Homo sapiens GN=RBP4 PE=1 SV=3 - [RET4_HUMAN] | 79.60 | 2 | 15 | 15 | 3180 | 921.3 | 130.4 |
| P02760 | Protein AMBP OS=Homo sapiens GN=AMBP PE=1 SV=1 - [AMBP_HUMAN] | 50.85 | 2 | 17 | 17 | 4839 | 1639.5 | 199.9 |
| P02763 | Alpha-1-acid glycoprotein 1 OS=Homo sapiens GN=ORM1 PE=1 SV=1 - [A1AG1_HUMAN] | 59.70 | 1 | 9 | 18 | 7307 | 2916.4 | 343.9 |
| P02765 | Alpha-2-HS-glycoprotein OS=Homo sapiens GN=AHSG PE=1 SV=1 - [FETUA_HUMAN] | 61.04 | 2 | 18 | 18 | 6718 | 2356.7 | 261.1 |
| P02766 | Transthyretin OS=Homo sapiens GN=TTR PE=1 SV=1 - [TTHY_HUMAN] | 77.55 | 1 | 16 | 16 | 6144 | 2692.0 | 345.3 |
| P02768 | Serum albumin OS=Homo sapiens GN=ALB PE=1 SV=2 - [ALBU_HUMAN] | 94.91 | 1 | 122 | 122 | 246532 | 77105.7 | 10904.5 |
| P02774 | Vitamin D-binding protein OS=Homo sapiens GN=GC PE=1 SV=1 - [VTDB_HUMAN] | 80.38 | 1 | 8 | 41 | 14377 | 4200.8 | 600.7 |
| P02787 | Serotransferrin OS=Homo sapiens GN=TF PE=1 SV=3 - [TRFE_HUMAN] | 83.52 | 1 | 80 | 80 | 45597 | 13813.7 | 1835.8 |
| P02790 | Hemopexin OS=Homo sapiens GN=HPX PE=1 SV=2 - [HEMO_HUMAN] | 76.84 | 1 | 34 | 34 | 10281 | 3280.3 | 436.2 |
| P04003 | C4b-binding protein alpha chain OS=Homo sapiens GN=C4BPA PE=1 SV=2 - [C4BPA_HUMAN] | 66.67 | 1 | 31 | 31 | 5622 | 1332.0 | 214.5 |
| P04004 | Vitronectin OS=Homo sapiens GN=VTN PE=1 SV=1 - [VTNC_HUMAN] | 33.89 | 1 | 17 | 17 | 2943 | 635.2 | 103.6 |
| P04114 | Apolipoprotein B-100 OS=Homo sapiens GN=APOB PE=1 SV=2 - [APOB_HUMAN] | 67.98 | 1 | 299 | 299 | 45060 | 14039.4 | 1785.2 |
| P04196 | Histidine-rich glycoprotein OS=Homo sapiens GN=HRG PE=1 SV=1 - [HRG_HUMAN] | 62.67 | 1 | 24 | 24 | 4226 | 1297.0 | 196.5 |
| P04217 | Alpha-1B-glycoprotein OS=Homo sapiens GN=A1BG PE=1 SV=4 - [A1BG_HUMAN] | 51.72 | 1 | 17 | 17 | 4345 | 1513.1 | 209.2 |
| P05090 | Apolipoprotein D OS=Homo sapiens GN=APOD PE=1 SV=1 - [APOD_HUMAN] | 40.21 | 3 | 9 | 9 | 3260 | 1052.0 | 113.5 |
| P05155 | Plasma protease C1 inhibitor OS=Homo sapiens GN=SERPING1 PE=1 SV=2 - [IC1_HUMAN] | 41.00 | 5 | 23 | 23 | 5454 | 1802.0 | 228.7 |
| P05546 | Heparin cofactor 2 OS=Homo sapiens GN=SERPIND1 PE=1 SV=3 - [HEP2_HUMAN] | 54.51 | 1 | 22 | 22 | 3091 | 958.7 | 122.5 |
| P06331 | Ig heavy chain V-II region ARH-77 OS=Homo sapiens PE=4 SV=1 - [HV209_HUMAN] | 17.12 | 1 | 2 | 2 | 998 | 474.4 | 50.3 |
| P06396 | Gelsolin OS=Homo sapiens GN=GSN PE=1 SV=1 - [GELS_HUMAN] | 52.17 | 4 | 36 | 36 | 4883 | 1445.5 | 202.7 |
| P06727 | Apolipoprotein A-IV OS=Homo sapiens GN=APOA4 PE=1 SV=3 - [APOA4_HUMAN] | 80.30 | 1 | 38 | 38 | 12338 | 3451.2 | 513.9 |
| P07357 | Complement component C8 alpha chain OS=Homo sapiens GN=C8A PE=1 SV=2 - [CO8A_HUMAN] | 10.93 | 3 | 2 | 2 | 6 | 34.3 | 9.4 |
| P07477 | Trypsin-1 OS=Homo sapiens GN=PRSS1 PE=1 SV=1 - [TRY1_HUMAN] | 68.81 | 2 | 10 | 10 | 1205 | 387.8 | 55.3 |
| P08603 | Complement factor H OS=Homo sapiens GN=CFH PE=1 SV=4 - [CFAH_HUMAN] | 34.13 | 2 | 12 | 12 | 282 | 283.8 | 29.1 |
| P08697 | Alpha-2-antiplasmin OS=Homo sapiens GN=SERPINF2 PE=1 SV=3 - [A2AP_HUMAN] | 66.13 | 1 | 67 | 73 | 11682 | 3394.7 | 456.4 |
| P09871 | Complement C1s subcomponent OS=Homo sapiens GN=C1S PE=1 SV=1 - [C1S_HUMAN] | 16.70 | 1 | 6 | 6 | 18 | 360.8 | 42.3 |
| P0CG05 | Ig lambda-2 chain C regions OS=Homo sapiens GN=IGLC2 PE=1 SV=1 - [LAC2_HUMAN] | 65.08 | 1 | 4 | 93 | 13537 | 7010.4 | 497.3 |
| P10909 | Clusterin OS=Homo sapiens GN=CLU PE=1 SV=1 - [CLUS_HUMAN] | 16.19 | 2 | 4 | 4 | 22 | 93.5 | 11.9 |
| P13671 | Complement component C6 OS=Homo sapiens GN=C6 PE=1 SV=3 - [CO6_HUMAN] | 19.69 | 1 | 8 | 9 | 95 | 111.3 | 19.5 |
| P19652 | Alpha-1-acid glycoprotein 2 OS=Homo sapiens GN=ORM2 PE=1 SV=2 - [A1AG2_HUMAN] | 5.68 | 3 | 4 | 4 | 50 | 86.0 | 12.6 |
| P19823 | Inter-alpha-trypsin inhibitor heavy chain H2 OS=Homo sapiens GN=ITIH2 PE=1 SV=2 - [ITIH2_HUMAN] | 61.19 | 1 | 9 | 18 | 2917 | 826.3 | 127.2 |
| P19827 | Inter-alpha-trypsin inhibitor heavy chain H1 OS=Homo sapiens GN=ITIH1 PE=1 SV=3 - [ITIH1_HUMAN] | 45.45 | 2 | 39 | 39 | 9465 | 2699.8 | 349.4 |
| P25311 | Zinc-alpha-2-glycoprotein OS=Homo sapiens GN=AZGP1 PE=1 SV=2 - [ZA2G_HUMAN] | 14.84 | 2 | 2 | 2 | 4 | 92.7 | 13.3 |
| P27169 | Serum paraoxonase/arylesterase 1 OS=Homo sapiens GN=PON1 PE=1 SV=3 - [PON1_HUMAN] | 6.89 | 2 | 5 | 5 | 30 | 73.8 | 15.3 |
| P36955 | Pigment epithelium-derived factor OS=Homo sapiens GN=SERPINF1 PE=1 SV=4 - [PEDF_HUMAN] | 9.66 | 2 | 4 | 4 | 13 | 200.6 | 12.2 |
| P43652 | Afamin OS=Homo sapiens GN=AFM PE=1 SV=1 - [AFAM_HUMAN] | 17.86 | 5 | 8 | 8 | 103 | 140.8 | 14.3 |
| P68871 | Hemoglobin subunit beta OS=Homo sapiens GN=HBB PE=1 SV=2 - [HBB_HUMAN] | 4.33 | 2 | 2 | 2 | 4 | 56.4 | 7.8 |
| P69905 | Hemoglobin subunit alpha OS=Homo sapiens GN=HBA1 PE=1 SV=2 - [HBA_HUMAN] | 95.24 | 1 | 8 | 14 | 4431 | 1936.2 | 200.0 |
| P80748 | Ig lambda chain V-III region LOI OS=Homo sapiens PE=1 SV=1 - [LV302_HUMAN] | 48.91 | 1 | 5 | 5 | 16 | 206.0 | 22.5 |
| P01764 | Ig heavy chain V-III region VH26 OS=Homo sapiens PE=1 SV=1 - [HV303_HUMAN] | 18.80 | 1 | 2 | 2 | 611 | 485.1 | 53.6 |
| P02735; P0DJI8 | Serum amyloid A protein OS=Homo sapiens GN=SAA1 PE=1 SV=2 - [SAA_HUMAN] | 72.13 | 5 | 5 | 11 | 2562 | 606.7 | 113.3 |
| P00450 | Ceruloplasmin OS=Homo sapiens GN=CP PE=1 SV=1 - [CERU_HUMAN] | 67.04 | 1 | 53 | 63 | 11520 | 3503.0 | 491.6 |
| P01591 | Immunoglobulin J chain OS=Homo sapiens GN=IGJ PE=1 SV=4 - [IGJ_HUMAN] | 64.15 | 4 | 7 | 7 | 659 | 193.0 | 25.4 |
| P01625 | Ig kappa chain V-IV region Len OS=Homo sapiens PE=1 SV=2 - [KV402_HUMAN] | 40.35 | 1 | 3 | 4 | 1329 | 715.6 | 66.5 |
| P01717 | Ig lambda chain V-IV region Hil OS=Homo sapiens PE=1 SV=1 - [LV403_HUMAN] | 28.04 | 1 | 2 | 2 | 125 | 211.9 | 31.4 |
| P01742 | Ig heavy chain V-I region EU OS=Homo sapiens PE=1 SV=1 - [HV101_HUMAN] | 16.24 | 1 | 2 | 2 | 535 | 317.9 | 35.1 |
| P01743 | Ig heavy chain V-I region HG3 OS=Homo sapiens PE=4 SV=1 - [HV102_HUMAN] | 28.21 | 1 | 2 | 3 | 822 | 182.1 | 34.6 |
| P02751 | Fibronectin OS=Homo sapiens GN=FN1 PE=1 SV=4 - [FINC_HUMAN] | 38.26 | 16 | 64 | 64 | 2197 | 555.1 | 89.1 |
| P03952 | Plasma kallikrein OS=Homo sapiens GN=KLKB1 PE=1 SV=1 - [KLKB1_HUMAN] | 53.92 | 3 | 31 | 31 | 2201 | 595.8 | 79.3 |
| P05156 | Complement factor I OS=Homo sapiens GN=CFI PE=1 SV=2 - [CFAI_HUMAN] | 47.00 | 4 | 27 | 27 | 1463 | 542.4 | 61.4 |
| P07225 | Vitamin K-dependent protein S OS=Homo sapiens GN=PROS1 PE=1 SV=1 - [PROS_HUMAN] | 8.68 | 1 | 3 | 3 | 16 | 97.5 | 11.9 |
| P07358 | Complement component C8 beta chain OS=Homo sapiens GN=C8B PE=1 SV=3 - [CO8B_HUMAN] | 47.95 | 1 | 23 | 23 | 1844 | 618.4 | 71.7 |
| P08185 | Corticosteroid-binding globulin OS=Homo sapiens GN=SERPINA6 PE=1 SV=1 - [CBG_HUMAN] | 16.75 | 1 | 14 | 14 | 67 | 106.9 | 13.1 |
| P20851 | C4b-binding protein beta chain OS=Homo sapiens GN=C4BPB PE=1 SV=1 - [C4BPB_HUMAN] | 42.98 | 1 | 40 | 51 | 10655 | 3825.3 | 458.7 |
| P22792 | Carboxypeptidase N subunit 2 OS=Homo sapiens GN=CPN2 PE=1 SV=3 - [CPN2_HUMAN] | 9.71 | 4 | 21 | 21 | 128 | 188.3 | 26.1 |
| P35542 | Serum amyloid A-4 protein OS=Homo sapiens GN=SAA4 PE=1 SV=2 - [SAA4_HUMAN] | 34.67 | 1 | 13 | 14 | 65 | 303.6 | 27.4 |
| P51884 | Lumican OS=Homo sapiens GN=LUM PE=1 SV=2 - [LUM_HUMAN] | 10.24 | 2 | 5 | 5 | 59 | 53.9 | 7.7 |
| P01703 | Ig lambda chain V-I region NEWM OS=Homo sapiens PE=1 SV=1 - [LV105_HUMAN] | 33.98 | 1 | 3 | 3 | 175 | 136.6 | 13.6 |
| P01877 | Ig alpha-2 chain C region OS=Homo sapiens GN=IGHA2 PE=1 SV=3 - [IGHA2_HUMAN] | 69.41 | 1 | 8 | 19 | 6716 | 2374.4 | 342.0 |
| O14791 | Apolipoprotein L1 OS=Homo sapiens GN=APOL1 PE=1 SV=5 - [APOL1_HUMAN] | 33.92 | 4 | 18 | 18 | 1115 | 263.5 | 44.3 |
| P01042 | Kininogen-1 OS=Homo sapiens GN=KNG1 PE=1 SV=2 - [KNG1_HUMAN] | 57.61 | 1 | 15 | 44 | 8900 | 2838.3 | 357.5 |
| P02747 | Complement C1q subcomponent subunit C OS=Homo sapiens GN=C1QC PE=1 SV=3 - [C1QC_HUMAN] | 25.31 | 1 | 5 | 5 | 706 | 226.7 | 31.3 |
| P04433 | Ig kappa chain V-III region VG (Fragment) OS=Homo sapiens PE=1 SV=1 - [KV309_HUMAN] | 37.39 | 1 | 3 | 5 | 498 | 105.4 | 20.6 |
| P07360 | Complement component C8 gamma chain OS=Homo sapiens GN=C8G PE=1 SV=3 - [CO8G_HUMAN] | 7.03 | 2 | 3 | 3 | 12 | 89.1 | 11.8 |
| P10643 | Complement component C7 OS=Homo sapiens GN=C7 PE=1 SV=2 - [CO7_HUMAN] | 23.74 | 1 | 3 | 6 | 16 | 233.5 | 25.5 |
| Q06033 | Inter-alpha-trypsin inhibitor heavy chain H3 OS=Homo sapiens GN=ITIH3 PE=1 SV=2 - [ITIH3_HUMAN] | 14.05 | 2 | 7 | 7 | 194 | 195.0 | 23.4 |
| P02746 | Complement C1q subcomponent subunit B OS=Homo sapiens GN=C1QB PE=1 SV=3 - [C1QB_HUMAN] | 34.78 | 3 | 7 | 7 | 904 | 395.3 | 44.1 |
| O75636 | Ficolin-3 OS=Homo sapiens GN=FCN3 PE=1 SV=2 - [FCN3_HUMAN] | 35.45 | 3 | 9 | 9 | 646 | 321.1 | 37.2 |
| P01768 | Ig heavy chain V-III region CAM OS=Homo sapiens PE=1 SV=1 - [HV307_HUMAN] | 40.98 | 1 | 5 | 7 | 1458 | 518.2 | no score |
| P01781 | Ig heavy chain V-III region GAL OS=Homo sapiens PE=1 SV=1 - [HV320_HUMAN] | 40.52 | 1 | 4 | 4 | 1165 | 273.2 | 44.6 |
| P01880 | Ig delta chain C region OS=Homo sapiens GN=IGHD PE=1 SV=2 - [IGHD_HUMAN] | 27.08 | 2 | 10 | 10 | 877 | 329.4 | 45.1 |
| P20742 | Pregnancy zone protein OS=Homo sapiens GN=PZP PE=1 SV=4 - [PZP_HUMAN] | 47.86 | 2 | 36 | 36 | 7935 | 2850.9 | 359.5 |
| P35858 | Insulin-like growth factor-binding protein complex acid labile subunit OS=Homo sapiens GN=IGFALS PE=1 SV=1 - [ALS_HUMAN] | 3.93 | 2 | 7 | 7 | 16 | 173.5 | 28.0 |
| P61769 | Beta-2-microglobulin OS=Homo sapiens GN=B2M PE=1 SV=1 - [B2MG_HUMAN] | 39.86 | 2 | 4 | 4 | 40 | 175.9 | 19.9 |
| Q96PD5 | N-acetylmuramoyl-L-alanine amidase OS=Homo sapiens GN=PGLYRP2 PE=1 SV=1 - [PGRP2_HUMAN] | 32.47 | 2 | 11 | 11 | 943 | 432.2 | 49.4 |
| E9PFZ2 | Ceruloplasmin OS=Homo sapiens GN=CP PE=4 SV=1 - [E9PFZ2_HUMAN] | 59.94 | 1 | 40 | 50 | 826 | no score | 161.4 |
| P01042-2 | Isoform LMW of Kininogen-1 OS=Homo sapiens GN=KNG1 - [KNG1_HUMAN] | 72.13 | 2 | 5 | 34 | 4192 | no score | 348.6 |
| A0M8Q6 | Ig lambda-7 chain C region OS=Homo sapiens GN=IGLC7 PE=1 SV=2 - [LAC7_HUMAN] | 69.81 | 1 | 6 | 10 | 6230 | 2596.2 | 329.6 |
| P00740 | Coagulation factor IX OS=Homo sapiens GN=F9 PE=1 SV=2 - [FA9_HUMAN] | 35.57 | 2 | 12 | 13 | 492 | 295.7 | 38.4 |
| P02745 | Complement C1q subcomponent subunit A OS=Homo sapiens GN=C1QA PE=1 SV=2 - [C1QA_HUMAN] | 24.49 | 1 | 4 | 4 | 271 | 164.0 | 26.1 |
| P08519 | Apolipoprotein(a) OS=Homo sapiens GN=LPA PE=1 SV=1 - [APOA_HUMAN] | 12.08 | 1 | 2 | 2 | 24 | 211.6 | 19.4 |
| P29622 | Kallistatin OS=Homo sapiens GN=SERPINA4 PE=1 SV=3 - [KAIN_HUMAN] | 3.85 | 3 | 3 | 3 | 8 | 138.8 | 11.5 |
| Q14520 | Hyaluronan-binding protein 2 OS=Homo sapiens GN=HABP2 PE=1 SV=1 - [HABP2_HUMAN] | 11.30 | 1 | 3 | 3 | 13 | 75.8 | 13.7 |
| P00739 | Haptoglobin-related protein OS=Homo sapiens GN=HPR PE=1 SV=2 - [HPTR_HUMAN] | 67.24 | 2 | 12 | 30 | 19628 | 6210.0 | 839.4 |
| P05160 | Coagulation factor XIII B chain OS=Homo sapiens GN=F13B PE=1 SV=3 - [F13B_HUMAN] | 43.87 | 1 | 23 | 23 | 775 | 328.7 | 41.1 |
| P05452 | Tetranectin OS=Homo sapiens GN=CLEC3B PE=1 SV=3 - [TETN_HUMAN] | 39.60 | 2 | 8 | 8 | 571 | 213.3 | 29.2 |
| P00736 | Complement C1r subcomponent OS=Homo sapiens GN=C1R PE=1 SV=2 - [C1R_HUMAN] | 62.55 | 1 | 22 | 37 | 2792 | 898.2 | 125.0 |
| P00742 | Coagulation factor X OS=Homo sapiens GN=F10 PE=1 SV=2 - [FA10_HUMAN] | 29.51 | 3 | 13 | 14 | 470 | 178.3 | 25.0 |
| P01611 | Ig kappa chain V-I region Wes OS=Homo sapiens PE=1 SV=1 - [KV119_HUMAN] | 37.04 | 1 | 2 | 3 | 326 | 376.5 | 34.7 |
| P04264 | Keratin, type II cytoskeletal 1 OS=Homo sapiens GN=KRT1 PE=1 SV=6 - [K2C1_HUMAN] | 52.33 | 1 | 26 | 29 | 489 | 282.0 | 31.9 |
| A6NJ16 | Putative V-set and immunoglobulin domain-containing protein 6 OS=Homo sapiens GN=VSIG6 PE=5 SV=2 - [VSIG6_HUMAN] | 21.95 | 1 | 4 | 5 | 192 | 150.8 | 15.2 |
| D6RF35 | Vitamin D-binding protein OS=Homo sapiens GN=GC PE=4 SV=1 - [D6RF35_HUMAN] | 74.16 | 2 | 5 | 38 | 6306 | no score | 603.9 |
| P60709 | Actin, cytoplasmic 1 OS=Homo sapiens GN=ACTB PE=1 SV=1 - [ACTB_HUMAN] | 11.36 | 4 | 5 | 5 | 46 | 147.1 | 13.9 |
| O95445 | Apolipoprotein M OS=Homo sapiens GN=APOM PE=1 SV=2 - [APOM_HUMAN] | 42.55 | 3 | 10 | 10 | 345 | 106.4 | 16.8 |
| P01702 | Ig lambda chain V-I region NIG-64 OS=Homo sapiens PE=1 SV=1 - [LV104_HUMAN] | 14.41 | 2 | 3 | 3 | 7 | no score | 11.8 |
| P00748 | Coagulation factor XII OS=Homo sapiens GN=F12 PE=1 SV=3 - [FA12_HUMAN] | 38.21 | 1 | 20 | 20 | 673 | 283.7 | 43.9 |
| P05543 | Thyroxine-binding globulin OS=Homo sapiens GN=SERPINA7 PE=1 SV=2 - [THBG_HUMAN] | 47.47 | 1 | 16 | 16 | 916 | 331.7 | 47.0 |
| O75882 | Attractin OS=Homo sapiens GN=ATRN PE=1 SV=2 - [ATRN_HUMAN] | 16.17 | 3 | 22 | 22 | 641 | 234.6 | 35.5 |
| P01034 | Cystatin-C OS=Homo sapiens GN=CST3 PE=1 SV=1 - [CYTC_HUMAN] | 56.16 | 1 | 7 | 7 | 397 | 300.7 | 34.2 |
| P01714 | Ig lambda chain V-III region SH OS=Homo sapiens PE=1 SV=1 - [LV301_HUMAN] | 42.59 | 1 | 4 | 4 | 318 | 139.3 | 24.4 |
| P36980 | Complement factor H-related protein 2 OS=Homo sapiens GN=CFHR2 PE=1 SV=1 - [FHR2_HUMAN] | 56.46 | 1 | 24 | 24 | 3021 | 1048.0 | 120.5 |
| P18428 | Lipopolysaccharide-binding protein OS=Homo sapiens GN=LBP PE=1 SV=3 - [LBP_HUMAN] | 10.85 | 3 | 11 | 11 | 81 | 240.7 | 22.3 |
| P04211 | Ig lambda chain V region 4A OS=Homo sapiens PE=4 SV=1 - [LV001_HUMAN] | 21.37 | 1 | 3 | 3 | 114 | 174.2 | 18.0 |
| P01769 | Ig heavy chain V-III region GA OS=Homo sapiens PE=1 SV=1 - [HV308_HUMAN] | 24.59 | 1 | 2 | 2 | 219 | 320.4 | no score |
| P23142 | Fibulin-1 OS=Homo sapiens GN=FBLN1 PE=1 SV=4 - [FBLN1_HUMAN] | 38.46 | 1 | 3 | 4 | 274 | 139.9 | 20.3 |
| Q96IY4 | Carboxypeptidase B2 OS=Homo sapiens GN=CPB2 PE=1 SV=2 - [CBPB2_HUMAN] | 34.75 | 2 | 11 | 11 | 403 | 152.0 | 19.5 |
| P01617 | Ig kappa chain V-II region TEW OS=Homo sapiens PE=1 SV=1 - [KV204_HUMAN] | 38.94 | 3 | 2 | 4 | 965 | 498.9 | 56.6 |
| P02775 | Platelet basic protein OS=Homo sapiens GN=PPBP PE=1 SV=3 - [CXCL7_HUMAN] | 44.53 | 1 | 6 | 6 | 247 | 150.9 | 19.3 |
| Q9NZP8 | Complement C1r subcomponent-like protein OS=Homo sapiens GN=C1RL PE=1 SV=2 - [C1RL_HUMAN] | 16.63 | 2 | 6 | 8 | 273 | 157.8 | 17.3 |
| P00488 | Coagulation factor XIII A chain OS=Homo sapiens GN=F13A1 PE=1 SV=4 - [F13A_HUMAN] | 19.54 | 1 | 13 | 13 | 413 | 264.8 | 30.8 |
| P01767 | Ig heavy chain V-III region BUT OS=Homo sapiens PE=1 SV=1 - [HV306_HUMAN] | 35.65 | 1 | 2 | 3 | 554 | 392.4 | no score |
| P04208 | Ig lambda chain V-I region WAH OS=Homo sapiens PE=1 SV=1 - [LV106_HUMAN] | 42.20 | 1 | 4 | 4 | 59 | 83.8 | 12.7 |
| P00746 | Complement factor D OS=Homo sapiens GN=CFD PE=1 SV=5 - [CFAD_HUMAN] | 49.01 | 1 | 11 | 11 | 373 | 216.2 | 36.1 |
| P15169 | Carboxypeptidase N catalytic chain OS=Homo sapiens GN=CPN1 PE=1 SV=1 - [CBPN_HUMAN] | 11.98 | 3 | 2 | 2 | 6 | 167.2 | 13.3 |
| P27918 | Properdin OS=Homo sapiens GN=CFP PE=1 SV=2 - [PROP_HUMAN] | 5.52 | 1 | 2 | 2 | 6 | 110.1 | 12.4 |
| P01602 | Ig kappa chain V-I region HK102 (Fragment) OS=Homo sapiens GN=IGKV1-5 PE=4 SV=1 - [KV110_HUMAN] | 34.19 | 1 | 2 | 3 | 21 | 145.1 | 14.6 |
| P01609 | Ig kappa chain V-I region Scw OS=Homo sapiens PE=1 SV=1 - [KV117_HUMAN] | 42.59 | 1 | 2 | 3 | 1061 | 725.5 | 83.7 |
| P0DJI9 | Serum amyloid A-2 protein OS=Homo sapiens GN=SAA2 PE=1 SV=1 - [SAA2_HUMAN] | 93.40 | 1 | 5 | 11 | 11189 | 4101.6 | 495.3 |
| P80108 | Phosphatidylinositol-glycan-specific phospholipase D OS=Homo sapiens GN=GPLD1 PE=1 SV=3 - [PHLD_HUMAN] | 33.93 | 1 | 8 | 17 | 137 | 879.6 | 69.1 |
| Q9Y6R7 | IgGFc-binding protein OS=Homo sapiens GN=FCGBP PE=1 SV=3 - [FCGBP_HUMAN] | 10.45 | 1 | 22 | 22 | 307 | 231.5 | 24.8 |
| Q08380 | Galectin-3-binding protein OS=Homo sapiens GN=LGALS3BP PE=1 SV=1 - [LG3BP_HUMAN] | 1.06 | 1 | 4 | 4 | 8 | 81.8 | 8.3 |
| Q9UGM5 | Fetuin-B OS=Homo sapiens GN=FETUB PE=1 SV=2 - [FETUB_HUMAN] | 17.02 | 4 | 5 | 5 | 105 | 82.8 | 12.1 |
| B4E1Z4 | Complement factor B Ba fragment OS=Homo sapiens GN=CFB PE=2 SV=1 - [B4E1Z4_HUMAN] | 50.79 | 4 | 35 | 73 | 5504 | no score | 404.2 |
| P12259 | Coagulation factor V OS=Homo sapiens GN=F5 PE=1 SV=4 - [FA5_HUMAN] | 1.01 | 7 | 3 | 3 | 7 | 33.1 | 7.6 |
| P01619 | Ig kappa chain V-III region B6 OS=Homo sapiens PE=1 SV=1 - [KV301_HUMAN] | 58.33 | 1 | 3 | 5 | 2547 | 467.1 | 13.8 |
| P55056 | Apolipoprotein C-IV OS=Homo sapiens GN=APOC4 PE=1 SV=1 - [APOC4_HUMAN] | 3.91 | 5 | 1 | 2 | 7 | 194.3 | 16.2 |
| E7ETN3 | Uncharacterized protein OS=Homo sapiens PE=3 SV=1 - [E7ETN3_HUMAN] | 47.44 | 2 | 31 | 62 | 3056 | no score | 176.4 |
| P01610 | Ig kappa chain V-I region WEA OS=Homo sapiens PE=1 SV=1 - [KV118_HUMAN] | 37.04 | 1 | 2 | 4 | 1007 | 709.8 | 101.7 |
| P06889 | Ig lambda chain V-IV region MOL OS=Homo sapiens PE=1 SV=1 - [LV405_HUMAN] | 21.46 | 7 | 4 | 6 | 15 | no score | 27.0 |
| H0YFH3 | Complement C1r subcomponent OS=Homo sapiens GN=C1R PE=3 SV=1 - [H0YFH3_HUMAN] | 56.51 | 1 | 16 | 30 | 970 | no score | 60.5 |
| P04220 | Ig mu heavy chain disease protein OS=Homo sapiens PE=1 SV=1 - [MUCB_HUMAN] | 57.54 | 1 | 5 | 19 | 3938 | 2133.3 | 286.8 |
| P06312 | Ig kappa chain V-IV region (Fragment) OS=Homo sapiens GN=IGKV4-1 PE=4 SV=1 - [KV401_HUMAN] | 33.06 | 2 | 3 | 4 | 263 | no score | 29.9 |
| P06681 | Complement C2 OS=Homo sapiens GN=C2 PE=1 SV=2 - [CO2_HUMAN] | 34.44 | 6 | 6 | 28 | 1219 | 497.9 | 115.3 |
| Q13790 | Apolipoprotein F OS=Homo sapiens GN=APOF PE=1 SV=2 - [APOF_HUMAN] | 9.95 | 1 | 2 | 2 | 15 | 104.0 | 10.8 |
| Q92954 | Proteoglycan 4 OS=Homo sapiens GN=PRG4 PE=1 SV=2 - [PRG4_HUMAN] | 11.40 | 8 | 15 | 15 | 311 | 238.2 | 26.8 |
| P15814 | Immunoglobulin lambda-like polypeptide 1 OS=Homo sapiens GN=IGLL1 PE=1 SV=1 - [IGLL1_HUMAN] | 8.30 | 2 | 2 | 2 | 6 | 42.2 | 7.0 |
| P01605 | Ig kappa chain V-I region Lay OS=Homo sapiens PE=1 SV=1 - [KV113_HUMAN] | 31.48 | 1 | 2 | 3 | 202 | 78.9 | 29.8 |
| P04206 | Ig kappa chain V-III region GOL OS=Homo sapiens PE=1 SV=1 - [KV307_HUMAN] | 66.97 | 2 | 3 | 7 | 1095 | 703.0 | 80.0 |
| P07478 | Trypsin-2 OS=Homo sapiens GN=PRSS2 PE=1 SV=1 - [TRY2_HUMAN] | 33.60 | 2 | 5 | 5 | 4903 | 556.4 | 144.9 |
| Q03591 | Complement factor H-related protein 1 OS=Homo sapiens GN=CFHR1 PE=1 SV=2 - [FHR1_HUMAN] | 12.42 | 2 | 5 | 5 | 26 | 444.2 | 44.4 |
| Q04756 | Hepatocyte growth factor activator OS=Homo sapiens GN=HGFAC PE=1 SV=1 - [HGFA_HUMAN] | 50.00 | 2 | 2 | 13 | 1396 | 613.6 | 74.6 |
| P02776 | Platelet factor 4 OS=Homo sapiens GN=PF4 PE=1 SV=2 - [PLF4_HUMAN] | 35.64 | 1 | 4 | 4 | 142 | 147.8 | 27.5 |
| P04430 | Ig kappa chain V-I region BAN OS=Homo sapiens PE=1 SV=1 - [KV122_HUMAN] | 38.89 | 1 | 2 | 3 | 19 | 383.4 | 43.3 |
| P08571 | Monocyte differentiation antigen CD14 OS=Homo sapiens GN=CD14 PE=1 SV=2 - [CD14_HUMAN] | 40.33 | 1 | 21 | 21 | 438 | 204.7 | 28.1 |
| P01616 | Ig kappa chain V-II region MIL OS=Homo sapiens PE=1 SV=1 - [KV203_HUMAN] | 39.29 | 1 | 3 | 5 | 530 | 370.6 | 15.8 |
| P01621 | Ig kappa chain V-III region NG9 (Fragment) OS=Homo sapiens PE=1 SV=1 - [KV303_HUMAN] | 60.00 | 1 | 4 | 6 | 122 | no score | 24.4 |
| P01763 | Ig heavy chain V-III region WEA OS=Homo sapiens PE=1 SV=1 - [HV302_HUMAN] | 35.96 | 1 | 2 | 3 | 251 | 189.9 | 35.2 |
| P04434 | Ig kappa chain V-III region VH (Fragment) OS=Homo sapiens PE=4 SV=1 - [KV310_HUMAN] | 53.45 | 1 | 4 | 5 | 96 | 47.5 | 9.3 |
| P01608 | Ig kappa chain V-I region Roy OS=Homo sapiens PE=1 SV=1 - [KV116_HUMAN] | 24.07 | 1 | 1 | 2 | 683 | 411.2 | 64.6 |
| P01612 | Ig kappa chain V-I region Mev OS=Homo sapiens PE=1 SV=1 - [KV120_HUMAN] | 31.19 | 1 | 2 | 2 | 33 | 122.9 | 10.4 |
| P01613 | Ig kappa chain V-I region Ni OS=Homo sapiens PE=1 SV=1 - [KV121_HUMAN] | 30.36 | 1 | 1 | 2 | 329 | 331.9 | 38.0 |
| P05154 | Plasma serine protease inhibitor OS=Homo sapiens GN=SERPINA5 PE=1 SV=3 - [IPSP_HUMAN] | 26.11 | 4 | 9 | 9 | 141 | 154.7 | 18.2 |
| P07996 | Thrombospondin-1 OS=Homo sapiens GN=THBS1 PE=1 SV=2 - [TSP1_HUMAN] | 43.57 | 1 | 5 | 5 | 107 | 196.4 | 21.4 |
| P23142-4 | Isoform C of Fibulin-1 OS=Homo sapiens GN=FBLN1 - [FBLN1_HUMAN] | 35.70 | 3 | 11 | 17 | 374 | 140.2 | 30.6 |
| P41222 | Prostaglandin-H2 D-isomerase OS=Homo sapiens GN=PTGDS PE=1 SV=1 - [PTGDS_HUMAN] | 9.50 | 1 | 3 | 3 | 8 | 162.9 | 15.2 |
| Q14624 | Inter-alpha-trypsin inhibitor heavy chain H4 OS=Homo sapiens GN=ITIH4 PE=1 SV=4 - [ITIH4_HUMAN] | 10.94 | 1 | 1 | 5 | 62 | 797.0 | no score |
| Q16610 | Extracellular matrix protein 1 OS=Homo sapiens GN=ECM1 PE=1 SV=2 - [ECM1_HUMAN] | 17.62 | 1 | 3 | 3 | 22 | 133.2 | 14.2 |
| Q9UK55 | Protein Z-dependent protease inhibitor OS=Homo sapiens GN=SERPINA10 PE=1 SV=1 - [ZPI_HUMAN] | 31.31 | 2 | 12 | 12 | 162 | 104.0 | 16.0 |
| P43251 | Biotinidase OS=Homo sapiens GN=BTD PE=1 SV=2 - [BTD_HUMAN] | 3.41 | 2 | 2 | 2 | 6 | 53.3 | 12.0 |
| P01603 | Ig kappa chain V-I region Ka OS=Homo sapiens PE=1 SV=1 - [KV111_HUMAN] | 37.96 | 1 | 2 | 3 | 276 | 99.9 | 20.4 |
| P17936 | Insulin-like growth factor-binding protein 3 OS=Homo sapiens GN=IGFBP3 PE=1 SV=2 - [IBP3_HUMAN] | 25.66 | 1 | 4 | 7 | 22 | 238.8 | 33.2 |
| P04275 | von Willebrand factor OS=Homo sapiens GN=VWF PE=1 SV=4 - [VWF_HUMAN] | 11.06 | 1 | 25 | 25 | 406 | 315.5 | 37.3 |
| P06276 | Cholinesterase OS=Homo sapiens GN=BCHE PE=1 SV=1 - [CHLE_HUMAN] | 14.62 | 3 | 8 | 8 | 92 | 96.9 | 10.6 |
| P07737 | Profilin-1 OS=Homo sapiens GN=PFN1 PE=1 SV=2 - [PROF1_HUMAN] | 29.55 | 1 | 4 | 4 | 573 | 214.1 | 71.6 |
| P18065 | Insulin-like growth factor-binding protein 2 OS=Homo sapiens GN=IGFBP2 PE=1 SV=2 - [IBP2_HUMAN] | 20.27 | 5 | 6 | 6 | 160 | 186.4 | 16.7 |
| Q96KN2 | Beta-Ala-His dipeptidase OS=Homo sapiens GN=CNDP1 PE=1 SV=4 - [CNDP1_HUMAN] | 26.63 | 2 | 11 | 11 | 135 | 132.1 | 18.4 |
| P01701 | Ig lambda chain V-I region NEW OS=Homo sapiens PE=1 SV=1 - [LV103_HUMAN] | 60.36 | 1 | 4 | 4 | 102 | 64.2 | 11.9 |
| P01779 | Ig heavy chain V-III region TUR OS=Homo sapiens PE=1 SV=1 - [HV318_HUMAN] | 26.72 | 1 | 2 | 2 | 550 | 641.0 | 84.7 |
| P49908 | Selenoprotein P OS=Homo sapiens GN=SEPP1 PE=1 SV=3 - [SEPP1_HUMAN] | 4.23 | 1 | 2 | 2 | 3 | 129.5 | no score |
| P01709 | Ig lambda chain V-II region MGC OS=Homo sapiens PE=1 SV=1 - [LV206_HUMAN] | 18.92 | 2 | 3 | 3 | 16 | no score | 6.9 |
| P03951 | Coagulation factor XI OS=Homo sapiens GN=F11 PE=1 SV=1 - [FA11_HUMAN] | 18.24 | 4 | 11 | 11 | 72 | 109.5 | 13.0 |
| P13645 | Keratin, type I cytoskeletal 10 OS=Homo sapiens GN=KRT10 PE=1 SV=6 - [K1C10_HUMAN] | 8.00 | 1 | 2 | 2 | 4 | 103.0 | no score |
| Q6EMK4 | Vasorin OS=Homo sapiens GN=VASN PE=1 SV=1 - [VASN_HUMAN] | 6.39 | 1 | 3 | 3 | 75 | 188.2 | 20.5 |
| P11226 | Mannose-binding protein C OS=Homo sapiens GN=MBL2 PE=1 SV=2 - [MBL2_HUMAN] | 13.00 | 1 | 7 | 8 | 29 | 102.2 | 12.9 |
| P22891 | Vitamin K-dependent protein Z OS=Homo sapiens GN=PROZ PE=1 SV=2 - [PROZ_HUMAN] | 35.96 | 1 | 15 | 15 | 813 | 282.9 | 39.1 |
| P23083 | Ig heavy chain V-I region V35 OS=Homo sapiens PE=1 SV=1 - [HV103_HUMAN] | 15.00 | 2 | 4 | 4 | 34 | 103.7 | 10.3 |
| P35527 | Keratin, type I cytoskeletal 9 OS=Homo sapiens GN=KRT9 PE=1 SV=3 - [K1C9_HUMAN] | 8.27 | 2 | 5 | 5 | 15 | 50.6 | 9.7 |
| P62328 | Thymosin beta-4 OS=Homo sapiens GN=TMSB4X PE=1 SV=2 - [TYB4_HUMAN] | 65.55 | 3 | 6 | 6 | 641 | 403.3 | 35.3 |
| Q15582 | Transforming growth factor-beta-induced protein ig-h3 OS=Homo sapiens GN=TGFBI PE=1 SV=1 - [BGH3_HUMAN] | 16.29 | 1 | 4 | 4 | 12 | 142.9 | 15.6 |
| P01607 | Ig kappa chain V-I region Rei OS=Homo sapiens PE=1 SV=1 - [KV115_HUMAN] | 43.52 | 1 | 2 | 3 | 474 | 1256.5 | 122.3 |
| P01623 | Ig kappa chain V-III region WOL OS=Homo sapiens PE=1 SV=1 - [KV305_HUMAN] | 82.57 | 2 | 4 | 10 | 2400 | 898.8 | 122.9 |
| Q15848 | Adiponectin OS=Homo sapiens GN=ADIPOQ PE=1 SV=1 - [ADIPO_HUMAN] | 20.81 | 1 | 2 | 2 | 12 | 137.4 | 15.2 |
| Q99969 | Retinoic acid receptor responder protein 2 OS=Homo sapiens GN=RARRES2 PE=1 SV=1 - [RARR2_HUMAN] | 38.65 | 2 | 4 | 4 | 86 | 239.5 | 24.5 |
| Q9NPH3 | Interleukin-1 receptor accessory protein OS=Homo sapiens GN=IL1RAP PE=1 SV=2 - [IL1AP_HUMAN] | 2.98 | 3 | 2 | 2 | 60 | 155.7 | 13.9 |
| P01597 | Ig kappa chain V-I region DEE OS=Homo sapiens PE=1 SV=1 - [KV105_HUMAN] | 26.85 | 1 | 2 | 3 | 1707 | 923.3 | no score |
| P04209 | Ig lambda chain V-II region NIG-84 OS=Homo sapiens PE=1 SV=1 - [LV211_HUMAN] | 21.43 | 1 | 2 | 2 | 12 | 79.3 | 10.7 |
| P07359 | Platelet glycoprotein Ib alpha chain OS=Homo sapiens GN=GP1BA PE=1 SV=1 - [GP1BA_HUMAN] | 35.53 | 3 | 18 | 18 | 1393 | 408.5 | 63.7 |
| P16070 | CD44 antigen OS=Homo sapiens GN=CD44 PE=1 SV=3 - [CD44_HUMAN] | 15.96 | 1 | 2 | 2 | 189 | 90.7 | 22.8 |
| P35908 | Keratin, type II cytoskeletal 2 epidermal OS=Homo sapiens GN=KRT2 PE=1 SV=2 - [K22E_HUMAN] | 39.17 | 2 | 19 | 19 | 755 | 350.4 | 45.8 |
| Q6UXB8 | Peptidase inhibitor 16 OS=Homo sapiens GN=PI16 PE=1 SV=1 - [PI16_HUMAN] | 18.57 | 2 | 7 | 7 | 60 | 87.9 | 16.8 |
| P01620 | Ig kappa chain V-III region SIE OS=Homo sapiens PE=1 SV=1 - [KV302_HUMAN] | 71.56 | 2 | 2 | 7 | 513 | 706.3 | 81.5 |
| Q15323 | Keratin, type I cuticular Ha1 OS=Homo sapiens GN=KRT31 PE=2 SV=3 - [K1H1_HUMAN] | 13.28 | 3 | 3 | 5 | 201 | 133.3 | 15.3 |
| Q9Y490 | Talin-1 OS=Homo sapiens GN=TLN1 PE=1 SV=3 - [TLN1_HUMAN] | 10.47 | 2 | 26 | 27 | 204 | 209.7 | 25.8 |
| P01833 | Polymeric immunoglobulin receptor OS=Homo sapiens GN=PIGR PE=1 SV=4 - [PIGR_HUMAN] | 7.59 | 1 | 5 | 5 | 27 | 72.9 | 10.7 |
| P98160 | Basement membrane-specific heparan sulfate proteoglycan core protein OS=Homo sapiens GN=HSPG2 PE=1 SV=4 - [PGBM_HUMAN] | 45.95 | 1 | 4 | 4 | 1184 | 490.8 | 50.6 |
| Q12805 | EGF-containing fibulin-like extracellular matrix protein 1 OS=Homo sapiens GN=EFEMP1 PE=1 SV=2 - [FBLN3_HUMAN] | 9.94 | 2 | 3 | 3 | 13 | 67.0 | 10.8 |
| Q15828 | Cystatin-M OS=Homo sapiens GN=CST6 PE=1 SV=1 - [CYTM_HUMAN] | 7.61 | 2 | 5 | 5 | 96 | 181.6 | 23.8 |
| P01824 | Ig heavy chain V-II region WAH OS=Homo sapiens PE=1 SV=1 - [HV206_HUMAN] | 17.05 | 1 | 2 | 3 | 22 | 26.3 | 6.6 |
| P21333 | Filamin-A OS=Homo sapiens GN=FLNA PE=1 SV=4 - [FLNA_HUMAN] | 34.92 | 2 | 9 | 9 | 807 | 224.4 | 28.5 |
| P49747 | Cartilage oligomeric matrix protein OS=Homo sapiens GN=COMP PE=1 SV=2 - [COMP_HUMAN] | 15.93 | 4 | 3 | 8 | 35 | no score | 13.2 |
| P01615 | Ig kappa chain V-II region FR OS=Homo sapiens PE=1 SV=1 - [KV202_HUMAN] | 18.58 | 1 | 2 | 2 | 23 | 53.7 | 10.5 |
| P04207 | Ig kappa chain V-III region CLL OS=Homo sapiens PE=1 SV=2 - [KV308_HUMAN] | 45.74 | 1 | 2 | 5 | 263 | 79.3 | 22.2 |
| P08294 | Extracellular superoxide dismutase [Cu-Zn] OS=Homo sapiens GN=SOD3 PE=1 SV=2 - [SODE_HUMAN] | 22.85 | 1 | 4 | 4 | 15 | 221.0 | 28.0 |
| P18206 | Vinculin OS=Homo sapiens GN=VCL PE=1 SV=4 - [VINC_HUMAN] | 27.38 | 2 | 7 | 7 | 62 | 94.0 | 13.4 |
| E9PGN5 | Inter-alpha-trypsin inhibitor heavy chain H4 OS=Homo sapiens GN=ITIH4 PE=4 SV=1 - [E9PGN5_HUMAN] | 50.00 | 3 | 8 | 32 | 922 | no score | 146.8 |
| P00915 | Carbonic anhydrase 1 OS=Homo sapiens GN=CA1 PE=1 SV=2 - [CAH1_HUMAN] | 17.62 | 5 | 4 | 4 | 16 | 120.0 | 17.8 |
| P02144 | Myoglobin OS=Homo sapiens GN=MB PE=1 SV=2 - [MYG_HUMAN] | 35.06 | 5 | 7 | 7 | 36 | 85.2 | 12.7 |
| P02452 | Collagen alpha-1(I) chain OS=Homo sapiens GN=COL1A1 PE=1 SV=5 - [CO1A1_HUMAN] | 2.12 | 1 | 3 | 3 | 10 | 52.3 | 13.8 |
| P02786 | Transferrin receptor protein 1 OS=Homo sapiens GN=TFRC PE=1 SV=2 - [TFR1_HUMAN] | 8.29 | 3 | 6 | 6 | 61 | 184.8 | 20.4 |
| P04278 | Sex hormone-binding globulin OS=Homo sapiens GN=SHBG PE=1 SV=2 - [SHBG_HUMAN] | 20.65 | 9 | 6 | 6 | 26 | 106.8 | 12.2 |
| P04406 | Glyceraldehyde-3-phosphate dehydrogenase OS=Homo sapiens GN=GAPDH PE=1 SV=3 - [G3P_HUMAN] | 27.16 | 3 | 9 | 9 | 44 | 203.6 | 17.9 |
| P06311 | Ig kappa chain V-III region IARC/BL41 OS=Homo sapiens PE=1 SV=1 - [KV311_HUMAN] | 28.91 | 1 | 3 | 3 | 6 | 71.1 | 7.5 |
| P55058 | Phospholipid transfer protein OS=Homo sapiens GN=PLTP PE=1 SV=1 - [PLTP_HUMAN] | 37.01 | 1 | 7 | 7 | 117 | 82.6 | 12.1 |
| Q13103 | Secreted phosphoprotein 24 OS=Homo sapiens GN=SPP2 PE=1 SV=1 - [SPP24_HUMAN] | 3.17 | 1 | 2 | 2 | 3 | 96.0 | no score |
| P04075 | Fructose-bisphosphate aldolase A OS=Homo sapiens GN=ALDOA PE=1 SV=2 - [ALDOA_HUMAN] | 11.26 | 7 | 4 | 4 | 7 | 92.2 | 5.2 |
| P26927 | Hepatocyte growth factor-like protein OS=Homo sapiens GN=MST1 PE=1 SV=2 - [HGFL_HUMAN] | 5.37 | 6 | 3 | 3 | 18 | 193.3 | 32.2 |
| P48740 | Mannan-binding lectin serine protease 1 OS=Homo sapiens GN=MASP1 PE=1 SV=3 - [MASP1_HUMAN] | 62.60 | 1 | 39 | 39 | 5883 | 1535.6 | 199.5 |
| Q15166 | Serum paraoxonase/lactonase 3 OS=Homo sapiens GN=PON3 PE=1 SV=3 - [PON3_HUMAN] | 53.23 | 2 | 12 | 39 | 8122 | 2394.4 | 321.3 |
| P01773 | Ig heavy chain V-III region BUR OS=Homo sapiens PE=1 SV=1 - [HV312_HUMAN] | 15.97 | 1 | 2 | 2 | 6 | 93.9 | no score |
| P07195 | L-lactate dehydrogenase B chain OS=Homo sapiens GN=LDHB PE=1 SV=2 - [LDHB_HUMAN] | 31.13 | 1 | 2 | 3 | 37 | 73.5 | no score |
| P67936 | Tropomyosin alpha-4 chain OS=Homo sapiens GN=TPM4 PE=1 SV=3 - [TPM4_HUMAN] | 11.43 | 4 | 4 | 4 | 9 | 102.0 | 16.6 |
| Q9NSB4 | Keratin, type II cuticular Hb2 OS=Homo sapiens GN=KRT82 PE=1 SV=3 - [KRT82_HUMAN] | 7.41 | 1 | 2 | 5 | 63 | 417.4 | 42.3 |
| B7ZKJ8 | ITIH4 protein OS=Homo sapiens GN=ITIH4 PE=2 SV=1 - [B7ZKJ8_HUMAN] | 51.44 | 2 | 9 | 36 | 2096 | no score | 251.1 |
| P01776 | Ig heavy chain V-III region WAS OS=Homo sapiens PE=1 SV=1 - [HV315_HUMAN] | 23.93 | 1 | 2 | 2 | 52 | no score | 27.3 |
| P02042 | Hemoglobin subunit delta OS=Homo sapiens GN=HBD PE=1 SV=2 - [HBD_HUMAN] | 65.31 | 1 | 4 | 10 | 606 | 3763.0 | 347.4 |
| P06317 | Ig lambda chain V-VI region SUT OS=Homo sapiens PE=1 SV=1 - [LV603_HUMAN] | 27.93 | 1 | 3 | 3 | 11 | 42.1 | 7.9 |
| P19320 | Vascular cell adhesion protein 1 OS=Homo sapiens GN=VCAM1 PE=1 SV=1 - [VCAM1_HUMAN] | 28.69 | 1 | 14 | 14 | 585 | 398.6 | 44.3 |
| P32119 | Peroxiredoxin-2 OS=Homo sapiens GN=PRDX2 PE=1 SV=5 - [PRDX2_HUMAN] | 6.34 | 2 | 3 | 3 | 6 | 90.0 | 10.8 |
| P43121 | Cell surface glycoprotein MUC18 OS=Homo sapiens GN=MCAM PE=1 SV=2 - [MUC18_HUMAN] | 21.58 | 5 | 4 | 4 | 136 | 182.1 | 19.2 |
| P61626 | Lysozyme C OS=Homo sapiens GN=LYZ PE=1 SV=1 - [LYSC_HUMAN] | 9.78 | 5 | 2 | 2 | 8 | 98.4 | 12.8 |
| Q08830 | Fibrinogen-like protein 1 OS=Homo sapiens GN=FGL1 PE=1 SV=3 - [FGL1_HUMAN] | 23.25 | 1 | 14 | 14 | 342 | 186.7 | 25.7 |
| Q9BXR6 | Complement factor H-related protein 5 OS=Homo sapiens GN=CFHR5 PE=1 SV=1 - [FHR5_HUMAN] | 15.64 | 2 | 6 | 7 | 40 | 163.3 | 14.1 |
| E9PBV3 | Suprabasin OS=Homo sapiens GN=SBSN PE=4 SV=1 - [E9PBV3_HUMAN] | 20.00 | 2 | 4 | 4 | 13 | no score | 12.4 |
| P01624 | Ig kappa chain V-III region POM OS=Homo sapiens PE=1 SV=1 - [KV306_HUMAN] | 36.70 | 1 | 1 | 4 | 220 | 114.6 | 28.2 |
| P10645 | Chromogranin-A OS=Homo sapiens GN=CHGA PE=1 SV=7 - [CMGA_HUMAN] | 57.77 | 1 | 39 | 39 | 2018 | 541.4 | 83.4 |
| P23528 | Cofilin-1 OS=Homo sapiens GN=CFL1 PE=1 SV=3 - [COF1_HUMAN] | 28.11 | 2 | 7 | 13 | 124 | no score | 29.2 |
| P61224 | Ras-related protein Rap-1b OS=Homo sapiens GN=RAP1B PE=1 SV=1 - [RAP1B_HUMAN] | 48.80 | 17 | 16 | 16 | 742 | 285.9 | 39.8 |
| Q15485 | Ficolin-2 OS=Homo sapiens GN=FCN2 PE=1 SV=2 - [FCN2_HUMAN] | 34.62 | 3 | 10 | 14 | 156 | 593.3 | 62.8 |
| Q9HDC9 | Adipocyte plasma membrane-associated protein OS=Homo sapiens GN=APMAP PE=1 SV=2 - [APMAP_HUMAN] | 6.25 | 3 | 2 | 2 | 10 | 214.1 | 25.5 |
| G3V357 | Ribonuclease pancreatic OS=Homo sapiens GN=RNASE1 PE=3 SV=1 - [G3V357_HUMAN] | 26.72 | 2 | 2 | 2 | 6 | no score | 19.8 |
| P02788 | Lactotransferrin OS=Homo sapiens GN=LTF PE=1 SV=6 - [TRFL_HUMAN] | 18.03 | 4 | 11 | 11 | 53 | 252.4 | 31.1 |
| P05109 | Protein S100-A8 OS=Homo sapiens GN=S100A8 PE=1 SV=1 - [S10A8_HUMAN] | 72.04 | 1 | 7 | 7 | 86 | 411.7 | 52.0 |
| P08246 | Neutrophil elastase OS=Homo sapiens GN=ELANE PE=1 SV=1 - [ELNE_HUMAN] | 31.60 | 1 | 9 | 9 | 1298 | 511.2 | 62.2 |
| P0C0L4 | Complement C4-A OS=Homo sapiens GN=C4A PE=1 SV=1 - [CO4A_HUMAN] | 43.60 | 2 | 25 | 25 | 1878 | 745.6 | 90.6 |
| P0C0L5 | Complement C4-B OS=Homo sapiens GN=C4B PE=1 SV=1 - [CO4B_HUMAN] | 61.64 | 1 | 4 | 91 | 8770 | 6355.8 | 497.3 |
| O00187 | Mannan-binding lectin serine protease 2 OS=Homo sapiens GN=MASP2 PE=1 SV=4 - [MASP2_HUMAN] | 4.81 | 1 | 2 | 2 | 10 | 191.7 | 31.7 |
| O43790 | Keratin, type II cuticular Hb6 OS=Homo sapiens GN=KRT86 PE=1 SV=1 - [KRT86_HUMAN] | 27.16 | 2 | 10 | 16 | 139 | 29.0 | 7.4 |
| P11021 | 78 kDa glucose-regulated protein OS=Homo sapiens GN=HSPA5 PE=1 SV=2 - [GRP78_HUMAN] | 44.54 | 4 | 22 | 22 | 5053 | 1697.7 | 219.3 |
| P11597 | Cholesteryl ester transfer protein OS=Homo sapiens GN=CETP PE=1 SV=2 - [CETP_HUMAN] | 15.73 | 1 | 5 | 5 | 70 | 81.7 | 11.5 |
| P13591 | Neural cell adhesion molecule 1 OS=Homo sapiens GN=NCAM1 PE=1 SV=3 - [NCAM1_HUMAN] | 4.60 | 5 | 4 | 4 | 14 | 212.1 | 24.3 |
| P14618 | Pyruvate kinase isozymes M1/M2 OS=Homo sapiens GN=PKM2 PE=1 SV=4 - [KPYM_HUMAN] | 6.86 | 1 | 4 | 4 | 11 | 92.7 | 18.3 |
| P33908 | Mannosyl-oligosaccharide 1,2-alpha-mannosidase IA OS=Homo sapiens GN=MAN1A1 PE=1 SV=3 - [MA1A1_HUMAN] | 14.14 | 2 | 2 | 2 | 6 | 100.6 | 12.7 |
| P35579 | Myosin-9 OS=Homo sapiens GN=MYH9 PE=1 SV=4 - [MYH9_HUMAN] | 42.31 | 1 | 6 | 6 | 1000 | 211.3 | 35.0 |
| P78385 | Keratin, type II cuticular Hb3 OS=Homo sapiens GN=KRT83 PE=1 SV=2 - [KRT83_HUMAN] | 92.25 | 1 | 13 | 13 | 2088 | 967.3 | 100.1 |
| P78386 | Keratin, type II cuticular Hb5 OS=Homo sapiens GN=KRT85 PE=1 SV=1 - [KRT85_HUMAN] | 23.33 | 1 | 8 | 15 | 148 | 1003.4 | 110.5 |
| Q92820 | Gamma-glutamyl hydrolase OS=Homo sapiens GN=GGH PE=1 SV=2 - [GGH_HUMAN] | 7.86 | 1 | 2 | 2 | 6 | 72.4 | 12.6 |
| A2BHY4 | Complement component C4B (Childo blood group) OS=Homo sapiens GN=C4B-1 PE=4 SV=1 - [A2BHY4_HUMAN] | 67.37 | 4 | 5 | 98 | 11471 | no score | 886.9 |
| A6H8M8 | C4A protein OS=Homo sapiens GN=C4A PE=2 SV=1 - [A6H8M8_HUMAN] | 64.61 | 1 | 5 | 92 | 6186 | no score | 771.2 |
| B0V2C8 | Complement C4-A alpha chain OS=Homo sapiens GN=C4A PE=4 SV=1 - [B0V2C8_HUMAN] | 61.70 | 2 | 4 | 90 | 5200 | no score | 738.4 |
| F8VNV9 | Complement C4-B OS=Homo sapiens GN=C4B PE=4 SV=1 - [F8VNV9_HUMAN] | 66.55 | 1 | 5 | 94 | 6719 | no score | 1097.4 |
| P00558 | Phosphoglycerate kinase 1 OS=Homo sapiens GN=PGK1 PE=1 SV=3 - [PGK1_HUMAN] | 10.31 | 1 | 3 | 3 | 6 | 99.0 | 14.3 |
| P12814 | Alpha-actinin-1 OS=Homo sapiens GN=ACTN1 PE=1 SV=2 - [ACTN1_HUMAN] | 15.47 | 1 | 32 | 32 | 419 | 231.4 | 30.9 |
| P13598 | Intercellular adhesion molecule 2 OS=Homo sapiens GN=ICAM2 PE=1 SV=2 - [ICAM2_HUMAN] | 2.10 | 7 | 2 | 2 | 4 | 18.9 | 5.7 |
| P13796 | Plastin-2 OS=Homo sapiens GN=LCP1 PE=1 SV=6 - [PLSL_HUMAN] | 52.14 | 1 | 44 | 44 | 2583 | 602.0 | 98.9 |
| P15151 | Poliovirus receptor OS=Homo sapiens GN=PVR PE=1 SV=2 - [PVR_HUMAN] | 4.76 | 1 | 4 | 4 | 10 | 145.9 | 19.9 |
| P62937 | Peptidyl-prolyl cis-trans isomerase A OS=Homo sapiens GN=PPIA PE=1 SV=2 - [PPIA_HUMAN] | 45.45 | 1 | 3 | 3 | 6 | no score | 9.8 |
| Q01518 | Adenylyl cyclase-associated protein 1 OS=Homo sapiens GN=CAP1 PE=1 SV=5 - [CAP1_HUMAN] | 3.64 | 1 | 2 | 2 | 7 | 69.6 | 9.0 |
| Q13093 | Platelet-activating factor acetylhydrolase OS=Homo sapiens GN=PLA2G7 PE=1 SV=1 - [PAFA_HUMAN] | 17.24 | 5 | 6 | 6 | 44 | 138.2 | 13.2 |
| Q5JNX2 | Complement C4-A alpha chain OS=Homo sapiens GN=C4A PE=4 SV=1 - [Q5JNX2_HUMAN] | 13.15 | 3 | 7 | 7 | 112 | 99.6 | 13.9 |
| Q9NQ79 | Cartilage acidic protein 1 OS=Homo sapiens GN=CRTAC1 PE=1 SV=2 - [CRAC1_HUMAN] | 2.87 | 4 | 2 | 2 | 4 | 71.8 | 7.4 |
| Q9UHG3 | Prenylcysteine oxidase 1 OS=Homo sapiens GN=PCYOX1 PE=1 SV=3 - [PCYOX_HUMAN] | 8.91 | 3 | 3 | 3 | 12 | 161.4 | 15.9 |
| P04180 | Phosphatidylcholine-sterol acyltransferase OS=Homo sapiens GN=LCAT PE=1 SV=1 - [LCAT_HUMAN] | 8.18 | 3 | 3 | 3 | 12 | 71.9 | 12.2 |
| P26038 | Moesin OS=Homo sapiens GN=MSN PE=1 SV=3 - [MOES_HUMAN] | 60.07 | 1 | 22 | 22 | 2490 | 794.4 | 105.5 |
| P27797 | Calreticulin OS=Homo sapiens GN=CALR PE=1 SV=1 - [CALR_HUMAN] | 71.55 | 2 | 16 | 18 | 2433 | 927.8 | 102.6 |
| P48740-2 | Isoform 2 of Mannan-binding lectin serine protease 1 OS=Homo sapiens GN=MASP1 - [MASP1_HUMAN] | 25.04 | 3 | 8 | 13 | 89 | 150.6 | 21.9 |
| Q07954 | Prolow-density lipoprotein receptor-related protein 1 OS=Homo sapiens GN=LRP1 PE=1 SV=2 - [LRP1_HUMAN] | 41.35 | 3 | 30 | 30 | 2102 | 676.4 | 87.7 |
| Q14515 | SPARC-like protein 1 OS=Homo sapiens GN=SPARCL1 PE=1 SV=2 - [SPRL1_HUMAN] | 4.63 | 5 | 3 | 3 | 5 | no score | 8.6 |
| Q5VU59 | Tropomyosin 3 OS=Homo sapiens GN=TPM3 PE=2 SV=1 - [Q5VU59_HUMAN] | 57.11 | 2 | 4 | 84 | 5106 | no score | 721.2 |
| Q9Y4L1 | Hypoxia up-regulated protein 1 OS=Homo sapiens GN=HYOU1 PE=1 SV=1 - [HYOU1_HUMAN] | 2.00 | 2 | 2 | 2 | 20 | 182.2 | 20.8 |
| Q9Y6Z7 | Collectin-10 OS=Homo sapiens GN=COLEC10 PE=2 SV=2 - [COL10_HUMAN] | 9.39 | 1 | 2 | 2 | 4 | 88.9 | 10.7 |
| H7C1X1 | Uncharacterized protein OS=Homo sapiens PE=4 SV=1 - [H7C1X1_HUMAN] | 29.23 | 1 | 1 | 3 | 60 | no score | 20.7 |
| O00533 | Neural cell adhesion molecule L1-like protein OS=Homo sapiens GN=CHL1 PE=1 SV=4 - [CHL1_HUMAN] | 1.41 | 2 | 2 | 2 | 2 | no score | 3.8 |
| P01719 | Ig lambda chain V-V region DEL OS=Homo sapiens PE=1 SV=1 - [LV501_HUMAN] | 42.59 | 1 | 2 | 2 | 5 | 66.1 | 8.7 |
| P05062 | Fructose-bisphosphate aldolase B OS=Homo sapiens GN=ALDOB PE=1 SV=2 - [ALDOB_HUMAN] | 6.04 | 1 | 2 | 2 | 6 | 186.2 | 15.9 |
| P05164 | Myeloperoxidase OS=Homo sapiens GN=MPO PE=1 SV=1 - [PERM_HUMAN] | 7.65 | 3 | 6 | 6 | 21 | 140.9 | 31.4 |
| P06702 | Protein S100-A9 OS=Homo sapiens GN=S100A9 PE=1 SV=1 - [S10A9_HUMAN] | 70.18 | 1 | 6 | 6 | 48 | 721.3 | 92.0 |
| P06733 | Alpha-enolase OS=Homo sapiens GN=ENO1 PE=1 SV=2 - [ENOA_HUMAN] | 26.27 | 1 | 8 | 8 | 21 | 323.9 | 23.2 |
| P07307 | Asialoglycoprotein receptor 2 OS=Homo sapiens GN=ASGR2 PE=1 SV=2 - [ASGR2_HUMAN] | 42.90 | 2 | 24 | 24 | 1855 | 550.4 | 78.8 |
| P12111 | Collagen alpha-3(VI) chain OS=Homo sapiens GN=COL6A3 PE=1 SV=5 - [CO6A3_HUMAN] | 4.46 | 1 | 2 | 2 | 10 | 95.0 | 11.4 |
| P14625 | Endoplasmin OS=Homo sapiens GN=HSP90B1 PE=1 SV=1 - [ENPL_HUMAN] | 12.62 | 2 | 5 | 5 | 9 | 196.7 | 18.0 |
| P36222 | Chitinase-3-like protein 1 OS=Homo sapiens GN=CHI3L1 PE=1 SV=2 - [CH3L1_HUMAN] | 23.79 | 1 | 9 | 13 | 109 | 153.6 | 21.5 |
| P54652 | Heat shock-related 70 kDa protein 2 OS=Homo sapiens GN=HSPA2 PE=1 SV=1 - [HSP72_HUMAN] | 43.20 | 1 | 12 | 12 | 1176 | 352.6 | 52.8 |
| P80188 | Neutrophil gelatinase-associated lipocalin OS=Homo sapiens GN=LCN2 PE=1 SV=2 - [NGAL_HUMAN] | 25.48 | 1 | 17 | 17 | 309 | 228.1 | 25.2 |
| Q01459 | Di-N-acetylchitobiase OS=Homo sapiens GN=CTBS PE=1 SV=1 - [DIAC_HUMAN] | 1.00 | 1 | 3 | 3 | 16 | 101.6 | 13.9 |
| Q13822 | Ectonucleotide pyrophosphatase/phosphodiesterase family member 2 OS=Homo sapiens GN=ENPP2 PE=1 SV=3 - [ENPP2_HUMAN] | 15.64 | 2 | 4 | 4 | 104 | 171.3 | 21.9 |
| Q9ULV4 | Coronin-1C OS=Homo sapiens GN=CORO1C PE=1 SV=1 - [COR1C_HUMAN] | 5.06 | 3 | 2 | 2 | 6 | 48.2 | 10.2 |
| B0QYP8 | Beta-parvin (Fragment) OS=Homo sapiens GN=PARVB PE=4 SV=1 - [B0QYP8_HUMAN] | 11.34 | 4 | 2 | 2 | 2 | no score | 5.9 |
| F5H3P3 | Rho GDP-dissociation inhibitor 2 (Fragment) OS=Homo sapiens GN=ARHGDIB PE=4 SV=1 - [F5H3P3_HUMAN] | 9.43 | 3 | 2 | 2 | 2 | no score | 3.8 |
| H0YKX5 | Tropomyosin alpha-1 chain (Fragment) OS=Homo sapiens GN=TPM1 PE=4 SV=1 - [H0YKX5_HUMAN] | 20.42 | 8 | 2 | 4 | 6 | no score | 21.4 |
| O60814 | Histone H2B type 1-K OS=Homo sapiens GN=HIST1H2BK PE=1 SV=3 - [H2B1K_HUMAN] | 25.40 | 15 | 3 | 3 | 22 | 324.0 | 33.5 |
| O76013 | Keratin, type I cuticular Ha6 OS=Homo sapiens GN=KRT36 PE=1 SV=1 - [KRT36_HUMAN] | 12.42 | 2 | 3 | 6 | 64 | 789.6 | 82.1 |
| P01593 | Ig kappa chain V-I region AG OS=Homo sapiens PE=1 SV=1 - [KV101_HUMAN] | 41.67 | 1 | 1 | 4 | 2128 | 1059.6 | 107.7 |
| P02538 | Keratin, type II cytoskeletal 6A OS=Homo sapiens GN=KRT6A PE=1 SV=3 - [K2C6A_HUMAN] | 16.84 | 2 | 5 | 9 | 28 | 296.5 | 57.3 |
| P03973 | Antileukoproteinase OS=Homo sapiens GN=SLPI PE=1 SV=2 - [SLPI_HUMAN] | 10.61 | 1 | 2 | 2 | 4 | 54.6 | 6.0 |
| P04040 | Catalase OS=Homo sapiens GN=CAT PE=1 SV=3 - [CATA_HUMAN] | 6.26 | 1 | 3 | 3 | 10 | 226.6 | 22.0 |
| P08779 | Keratin, type I cytoskeletal 16 OS=Homo sapiens GN=KRT16 PE=1 SV=4 - [K1C16_HUMAN] | 49.69 | 3 | 20 | 20 | 1806 | 450.4 | 80.8 |
| P10412 | Histone H1.4 OS=Homo sapiens GN=HIST1H1E PE=1 SV=2 - [H14_HUMAN] | 66.39 | 4 | 5 | 11 | 719 | no score | 100.7 |
| P14780 | Matrix metalloproteinase-9 OS=Homo sapiens GN=MMP9 PE=1 SV=3 - [MMP9_HUMAN] | 1.87 | 1 | 2 | 2 | 2 | 30.1 | no score |
| P15144 | Aminopeptidase N OS=Homo sapiens GN=ANPEP PE=1 SV=4 - [AMPN_HUMAN] | 3.54 | 1 | 2 | 2 | 8 | 150.4 | 18.3 |
| P15153 | Ras-related C3 botulinum toxin substrate 2 OS=Homo sapiens GN=RAC2 PE=1 SV=1 - [RAC2_HUMAN] | 6.00 | 1 | 2 | 2 | 2 | 33.7 | no score |
| P15259 | Phosphoglycerate mutase 2 OS=Homo sapiens GN=PGAM2 PE=1 SV=3 - [PGAM2_HUMAN] | 29.48 | 1 | 10 | 10 | 392 | 362.1 | 39.9 |
| P16401 | Histone H1.5 OS=Homo sapiens GN=HIST1H1B PE=1 SV=3 - [H15_HUMAN] | 4.18 | 26 | 3 | 3 | 10 | 38.6 | 7.3 |
| P24158 | Myeloblastin OS=Homo sapiens GN=PRTN3 PE=1 SV=3 - [PRTN3_HUMAN] | 36.14 | 7 | 6 | 6 | 44 | 204.8 | 19.3 |
| P29401 | Transketolase OS=Homo sapiens GN=TKT PE=1 SV=3 - [TKT_HUMAN] | 22.81 | 3 | 9 | 9 | 194 | 104.7 | 18.3 |
| P30101 | Protein disulfide-isomerase A3 OS=Homo sapiens GN=PDIA3 PE=1 SV=4 - [PDIA3_HUMAN] | 53.40 | 1 | 20 | 20 | 1118 | 507.3 | 67.7 |
| P37837 | Transaldolase OS=Homo sapiens GN=TALDO1 PE=1 SV=2 - [TALDO_HUMAN] | 57.41 | 2 | 7 | 12 | 737 | 487.0 | 51.8 |
| P63104 | 14-3-3 protein zeta/delta OS=Homo sapiens GN=YWHAZ PE=1 SV=1 - [1433Z_HUMAN] | 21.21 | 3 | 4 | 4 | 8 | 135.8 | 8.6 |
| P68104 | Elongation factor 1-alpha 1 OS=Homo sapiens GN=EEF1A1 PE=1 SV=1 - [EF1A1_HUMAN] | 28.23 | 7 | 6 | 8 | 67 | 142.0 | 21.9 |
| P80511 | Protein S100-A12 OS=Homo sapiens GN=S100A12 PE=1 SV=2 - [S10AC_HUMAN] | 14.14 | 3 | 2 | 2 | 10 | 58.8 | 10.2 |
| Q14532 | Keratin, type I cuticular Ha2 OS=Homo sapiens GN=KRT32 PE=1 SV=3 - [K1H2_HUMAN] | 37.68 | 3 | 16 | 16 | 725 | 218.3 | 32.9 |
| Q8WZ42-3 | Isoform 3 of Titin OS=Homo sapiens GN=TTN - [TITIN_HUMAN] | 0.05 | 10 | 2 | 2 | 3 | no score | 7.2 |
| Q96KK5 | Histone H2A type 1-H OS=Homo sapiens GN=HIST1H2AH PE=1 SV=3 - [H2A1H_HUMAN] | 21.88 | 10 | 2 | 2 | 8 | 75.1 | 12.5 |
| Q9BTY2 | Plasma alpha-L-fucosidase OS=Homo sapiens GN=FUCA2 PE=1 SV=2 - [FUCO2_HUMAN] | 3.64 | 1 | 2 | 2 | 4 | 25.9 | 4.8 |
| B4DQP2 | Protein-arginine deiminase type-1 OS=Homo sapiens GN=PADI1 PE=2 SV=1 - [B4DQP2_HUMAN] | 9.70 | 4 | 2 | 2 | 2 | no score | 1.0 |

**Supplemental Table 2**: Spearman correlation analysis of 82 plasma proteins with changes of next-day C-reactive protein.

| Plasma protein | r | 95% CI | P |
| --- | --- | --- | --- |
| Ig_lambda_7_chain_C_region | 0,105 | -0.119 to 0.320 | 0.342 |
| Immunoglobulin_lambda_like_ polypeptide_ 5 | 0,181 | -0.031 to 0.378 | 0.085 |
| Ceruloplasmin | -0,045 | -0.263 to 0.176 | 0.679 |
| CD5_antigen_like | -0,097 | -0.303 to 0.116 | 0.358 |
| Ficolin_3 | -0,077 | -0.291 to 0.144 | 0.481 |
| Ceruloplasmin | -0,143 | -0.346 to 0.073 | 0.180 |
| Prothrombin | -0,345 | -0.519 to -0.143 | 0.0008 |
| Haptoglobin | -0,213 | -0.407 to -0.001 | 0.042 |
| Haptoglobin_related_protein | -0,068 | -0.286 to 0.157 | 0.543 |
| Coagulation_factor_IX | -0,143 | -0.354 to 0.081 | 0.195 |
| Plasminogen | -0,256 | -0.443 to -0.0467 | 0.014 |
| Antithrombin_III | -0,385 | -0.552 to -0.188 | 0.0002 |
| Alpha_1_antitrypsin | -0,277 | -0.462 to -0.069 | 0.007 |
| Alpha_1_antichymotrypsin | -0,367 | -0.537 to -0.168 | 0.0003 |
| Angiotensinogen | -0,405 | -0.568 to -0.211 | 0.0001 |
| Alpha_2_macroglobulin | -0,116 | -0.320 to 0.097 | 0.270 |
| Complement_C3 | -0,144 | -0.345 to 0.069 | 0.171 |
| Complement_C5 | -0,151 | -0.351 to 0.062 | 0.152 |
| Low_molecular_weight_kininogen_1 | -0,133 | -0.344 to 0.089 | 0.224 |
| Ig_kappa_chain_V_IV_region_Len | 0,134 | -0.082 to 0.338 | 0.209 |
| Ig_lambda_chain_V_I_region_NEW | 0,261 | 0.048 to 0.451 | 0.013 |
| Ig_lambda_chain_V_IV_region_Hil | 0,278 | 0.068 to 0.465 | 0.008 |
| Ig_heavy_chain_V_I_region_EU | 0,177 | -0.038 to 0.377 | 0.096 |
| Ig_heavy_chain_V_I_region_HG3 | 0,293 | 0.084 to 0.477 | 0.005 |
| Ig_heavy_chain_V_III_region_CAM | 0,164 | -0.056 to 0.370 | 0.131 |
| Ig_kappa_chain_C_region | 0,140 | -0.073 to 0.341 | 0.185 |
| Ig_gamma_1_chain_C_region | 0,213 | 0.001 to 0.407 | 0.042 |
| Ig_gamma_3_chain_C_region | 0,134 | -0.079 to 0.336 | 0.203 |
| Ig_mu_chain_C_region | -0,113 | -0.317 to 0.101 | 0.285 |
| Ig_alpha_1_chain_C_region | 0,271 | 0.063 to 0.457 | 0.009 |
| Ig_alpha_2_chain_C_region | 0,138 | -0.078 to 0.344 | 0.196 |
| Ig_delta_chain_C_region | 0,302 | 0.089 to 0.489 | 0.004 |
| Apolipoprotein_A_I | -0,113 | -0.317 to 0.100 | 0.283 |
| Apolipoprotein_C_I | 0,070 | -0.143 to 0.277 | 0.508 |
| Apolipoprotein_C_II | 0,114 | -0.100 to 0.318 | 0.280 |
| Apolipoprotein_C_III | 0,203 | -0.008 to 0.398 | 0.052 |
| Fibrinogen_alpha_chain | -0,163 | -0.362 to 0.050 | 0.121 |
| Serum_amyloid_P_component | 0,045 | -0.168 to 0.254 | 0.670 |
| Complement_component_C9 | -0,189 | -0.385 to 0.0236 | 0.072 |
| Leucine_rich_alpha_2_glycoprotein | 0,051 | -0.162 to 0.260 | 0.627 |
| Alpha_1_acid_glycoprotein_1 | -0,173 | -0.371 to 0.0399 | 0.100 |
| Alpha_2_HS_glycoprotein | -0,099 | -0.304 to 0.115 | 0.349 |
| Transthyretin | 0,084 | -0.129 to 0.291 | 0.424 |
| Serum_albumin | 0,235 | 0.025 to 0.426 | 0.024 |
| Serotransferrin | -0,108 | -0.313 to 0.105 | 0.303 |
| Hemopexin | -0,303 | -0.483 to -0.097 | 0.003 |
| Plasma_kallikrein | -0,069 | -0.279 to 0.146 | 0.515 |
| Vitronectin | -0,323 | -0.500 to -0.119 | 0.001 |
| Apolipoprotein_B_100 | -0,123 | -0.326 to 0.091 | 0.244 |
| Alpha_1B_glycoprotein | -0,255 | -0.443 to -0.046 | 0.014 |
| Apolipoprotein_D | 0,091 | -0.122 to 0.297 | 0.386 |
| Plasma_protease_C1_inhibitor | -0,320 | -0.498 to -0.116 | 0.001 |
| Complement_factor_I | -0,195 | -0.392 to 0.020 | 0.066 |
| Coagulation_factor_XIII_ B_chain | -0,183 | -0.390 to 0.041 | 0.098 |
| Tetranectin | 0.004 | -0.222 to 0.223 | 0.996 |
| Heparin_cofactor_2 | -0,173 | -0.371 to 0.039 | 0.100 |
| Ig_heavy_chain_V_II_region_ARH77 | 0,151 | -0.062 to 0.351 | 0.152 |
| Gelsolin | 0,284 | 0.076 to 0.467 | 0.006 |
| Apolipoprotein_A_IV | 0,108 | -0.105 to 0.313 | 0.305 |
| Complement_component_C8_alpha_chain | -0,222 | -0.414 to -0.010 | 0.034 |
| Complement_component_C8_beta_chain | -0,158 | -0.360 to 0.058 | 0.139 |
| Complement_component_C8_gamma_chain | -0,055 | -0.269 to 0.163 | 0.609 |
| Corticosteroid_binding_globulin | -0,226 | -0.420 to -0.012 | 0.032 |
| Alpha_2_antiplasmin | -0,368 | -0.538 to -0.169 | 0.0003 |
| Ig_lambda_2_chain_C_regions | 0,343 | 0.142 to 0.518 | 0.0008 |
| Clusterin | -0,120 | -0.324 to 0.093 | 0.253 |
| Complement_component_C6 | -0,301 | -0.482 to -0.095 | 0.003 |
| Alpha_1_acid_glycoprotein_2 | -0,214 | -0.407 to -0.002 | 0.041 |
| Inter_alpha_trypsin_inhibitor_heavy_chain | -0,275 | -0.460 to -0.067 | 0.008 |
| Inter_alpha_trypsin_inhibitor_heavy_chain_H1 | -0,133 | -0.336 to 0.080 | 0.206 |
| Pregnancy_zone_protein | -0,163 | -0.369 to 0.058 | 0.136 |
| C4b_binding_protein_beta_chain | -0,081 | -0.291 to 0.134 | 0.445 |
| Carboxypeptidase_N_subunit_2 | -0,153 | -0.355 to 0.063 | 0.151 |
| Serum_paraoxonase/arylesterase_1 | -0,050 | -0.259 to 0.162 | 0.632 |
| Serum_amyloid_A_4_protein | -0,136 | -0.341 to 0.079 | 0.201 |
| Insulin_like_growth_factor_binding_protein_complex_acid_labile_subunit | -0,138 | -0.347 to 0.082 | 0.205 |
| Lumican | 0,059 | -0.156 to 0.270 | 0.580 |
| Hemoglobin_subunit_beta | 0,159 | -0.054 to 0.358 | 0.132 |
| Hemoglobin_subunit_alpha | 0,112 | -0.101 to 0.316 | 0.288 |
| Ig_lambda_chain_V_III_region_LOI | 0,395 | 0.200 to 0.560 | 0.0001 |
| Inter_alpha_trypsin_inhibitor_heavy_ chain_H3 | -0,182 | -0.383 to 0.035 | 0.091 |
| Hyaluronan_binding_protein_2 | -0,267 | -0.461to -0.048 | 0.014 |

r indicates Spearman r. CI indicates confidence interval.
